# Supplementary figures and images for: Insights into adenosine A2A receptor activation through cooperative modulation of agonist and allosteric lipid interactions
Source: PLoS Comput Biol. 2020 Apr 16;16(4):e1007818. doi: 10.1371/journal.pcbi.1007818 (PMC7188303; doi:10.1371/journal.pcbi.1007818)

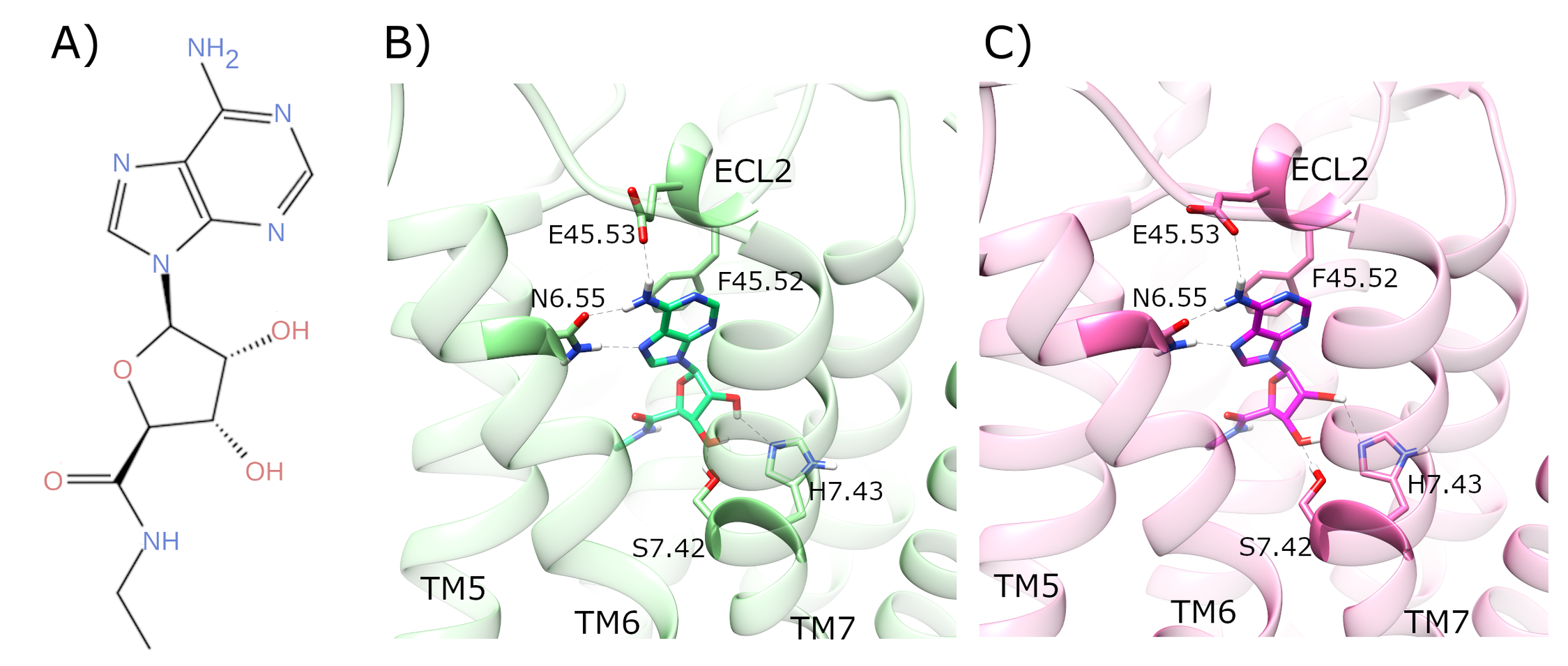

Supplement: S1 Fig — A) Molecular structure of NECA. Comparison of B) co-crystallized NECA (lime) in agonist-bound A2aR crystal structure (PDB entry: 2YDV, light green), and C) docked NECA (magenta) in the inactive crystal structure of A2aR (PDB entry: 4EIY, pink). Selected residues participating in ligand binding are displayed. Extracellular loop (ECL) 2 and transmembrane (TM) helices 5–7 are labelled. (TIF) [file pcbi.1007818.s002.tif]

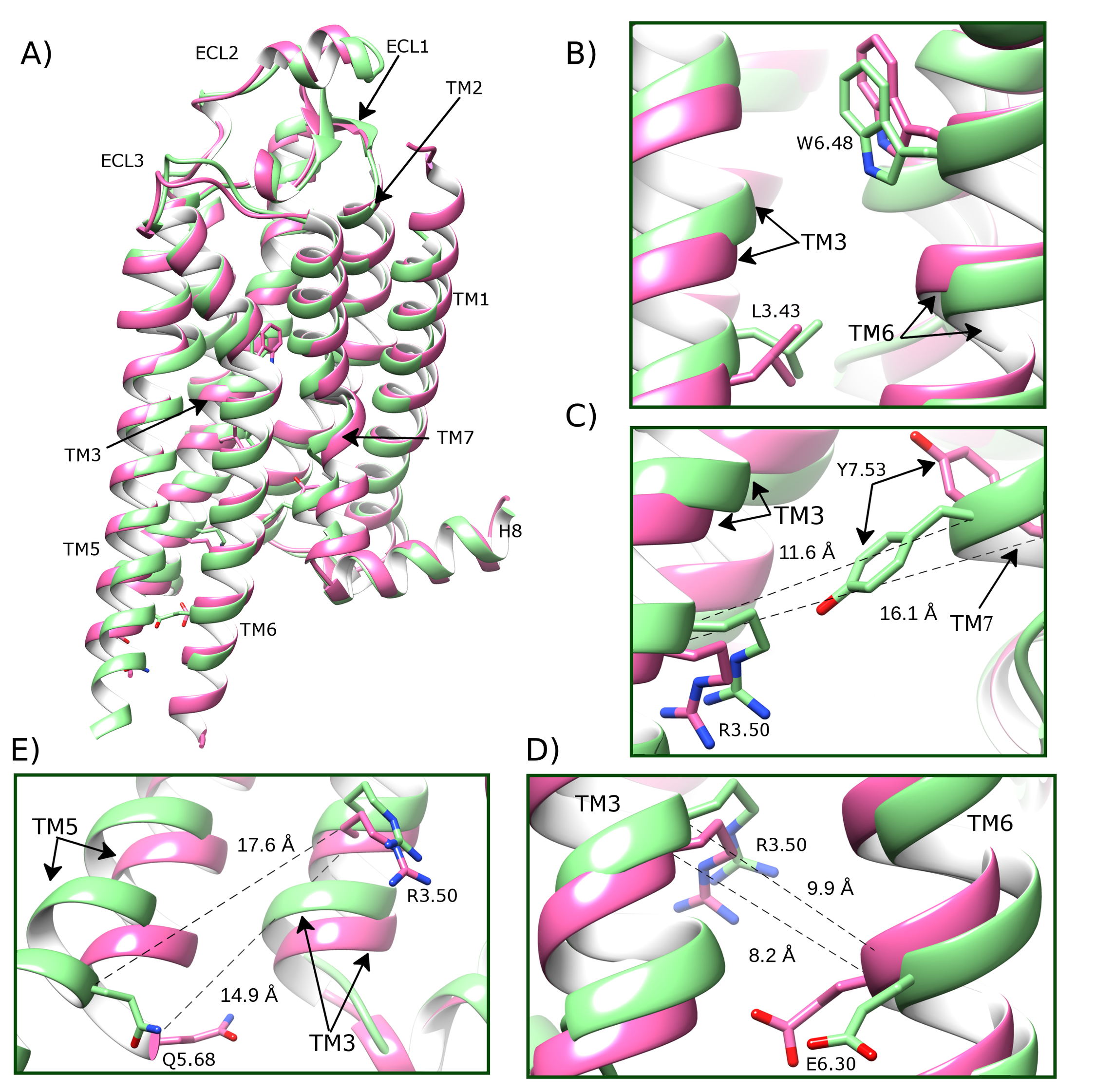

Supplement: S2 Fig — A) structural superposition of the intermediate adenosine-bound crystal structure (PDB entry: 2YDO, light green) on the inactive-state crystal structure (PDB entry: 4EIY, pink). B) Comparative positioning of residue L3.43 located on TM3 and rotameric state of W6.48 on TM6. C) Intracellular distance between residues R3.50 and Y7.53 on TM3 and TM7 (indicated by dashed lines) before/after receptor conformational change. D) Distance between residues R3.50 and Q5.68 (indicated by dashed lines). E) Partial separation of ionic-lock residues R3.50 and E6.30 on TM3 and TM6 (indicated by dashed lines). Relevant structural features are labelled: extracellular loops (ECL) 1, 2 and 3, and transmembrane (TM) helices 1–3, 5–7. (TIF) [file pcbi.1007818.s003.tif]

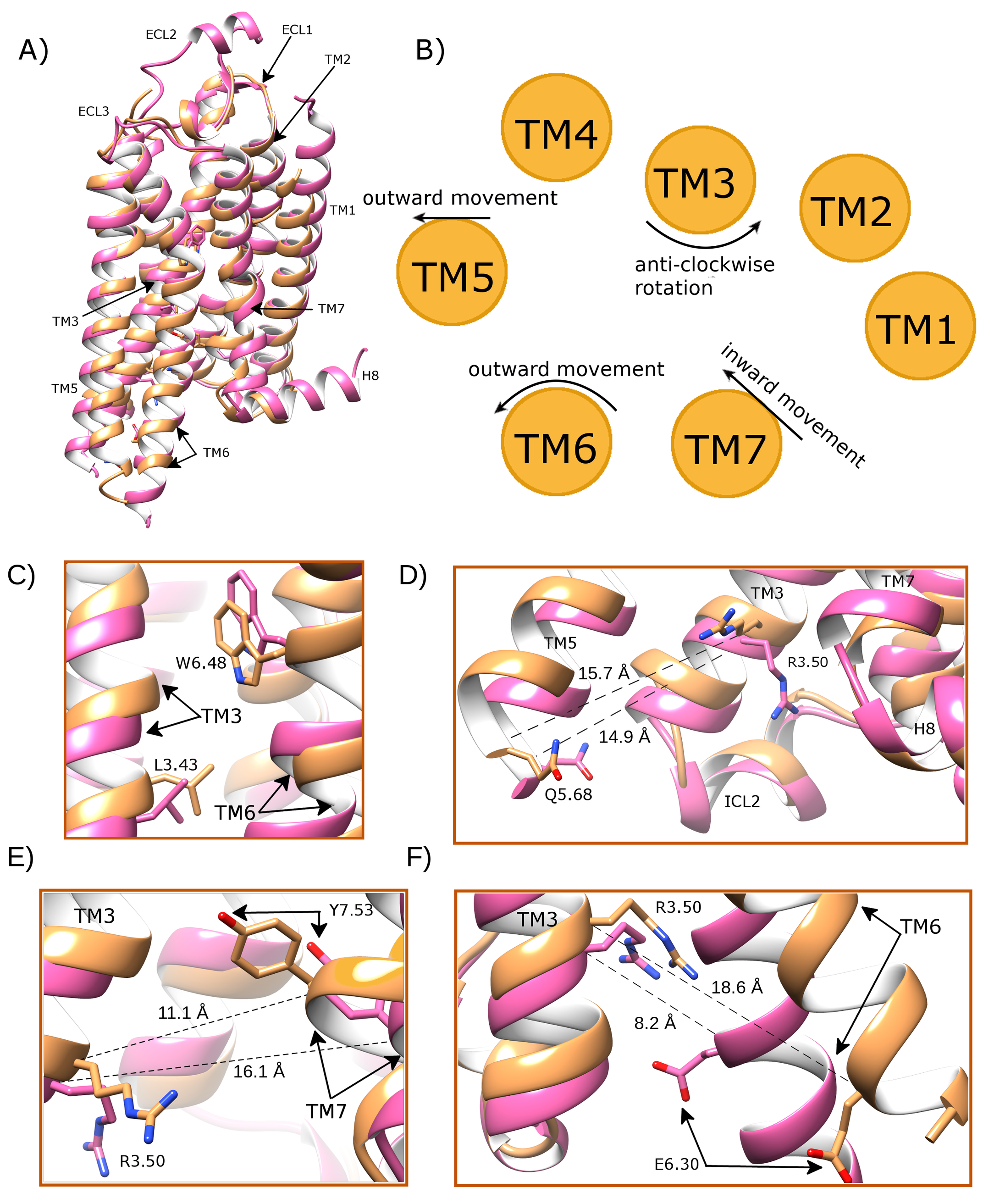

Supplement: S3 Fig — A) structural superposition of the active-state crystal structure (PDB entry: 6GDG, brown) on the inactive-state crystal structure (PDB entry: 4EIY, pink). B) Proposed scheme of activation for A2aR, including rotation and upwards axial movement of TM3, outwards movement of TM5, rotation plus outward movement of TM6, and inwards movement of TM7. C) Comparative positioning of residue L3.43 located on TM3 and rotameric state of W6.48 on TM6. D) Intracellular conformational change of TM5 with increased separation (indicated by dashed lines) between residues R3.50 and Q5.68 after receptor activation. E) Intracellular comparison of distance between residues R3.50 and Y7.53 after receptor activation (indicated by dashed lines). F) Intracellular conformational change of TM6 and separation (indicated by dashed lines) of ionic-lock residues R3.50 and E6.30 after receptor activation. Relevant structural features are labelled: intracellular loop (ICL) 2, extracellular loops (ECL) 1, 2 and 3, and transmembrane (TM) helices 1–3, 5–7. (TIF) [file pcbi.1007818.s004.tif]

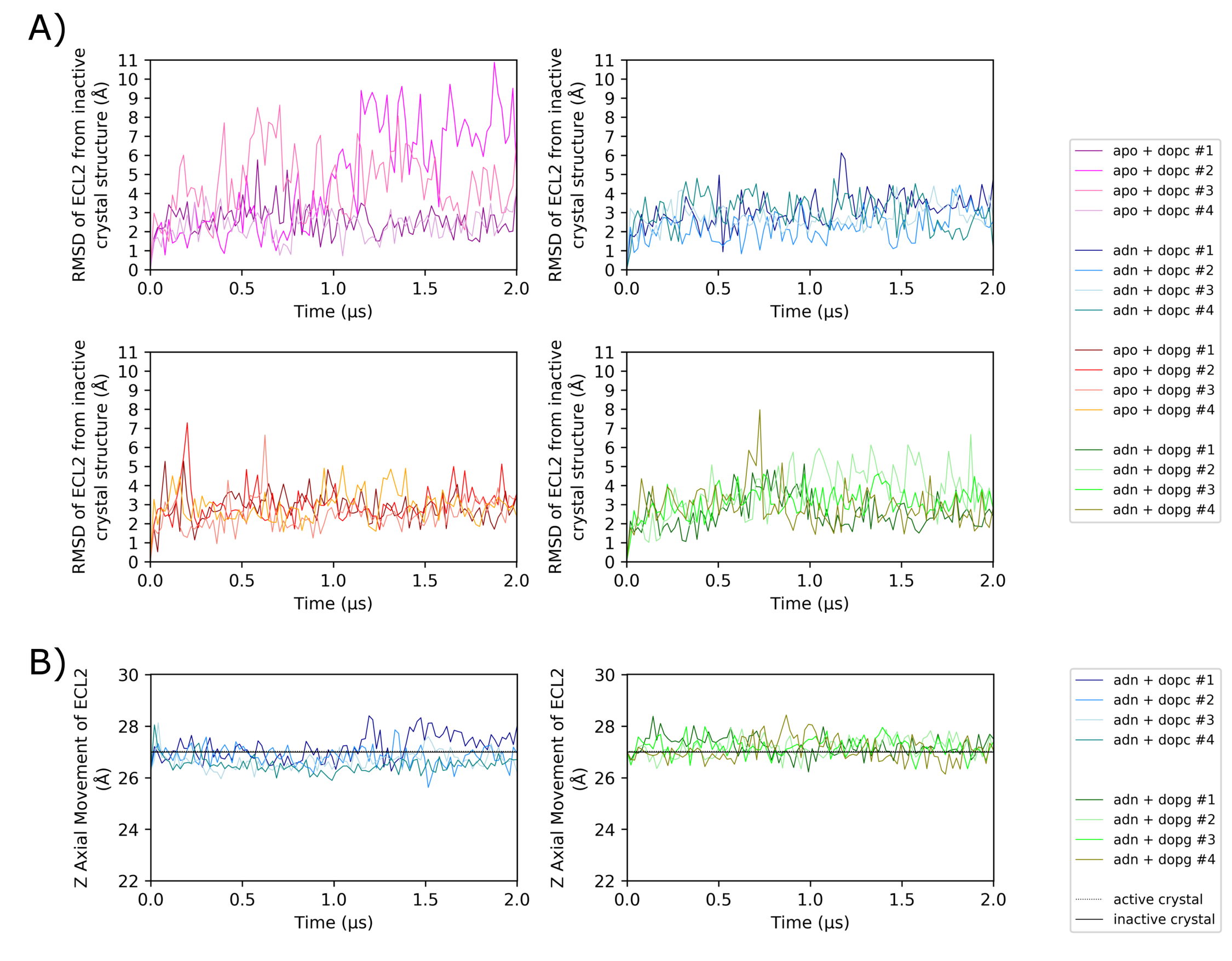

Supplement: S4 Fig — A) RMSD of ECL2 from the starting inactive A2aR crystal structure (PDB entry: 4EIY). B) Vertical movement of ECL2 along Z-axis (containing residues: G142-A173). MD simulations are performed in quadruplicate, with or without bound adenosine (ADN) in DOPC or DOPG homogeneous membranes. (TIF) [file pcbi.1007818.s005.tif]

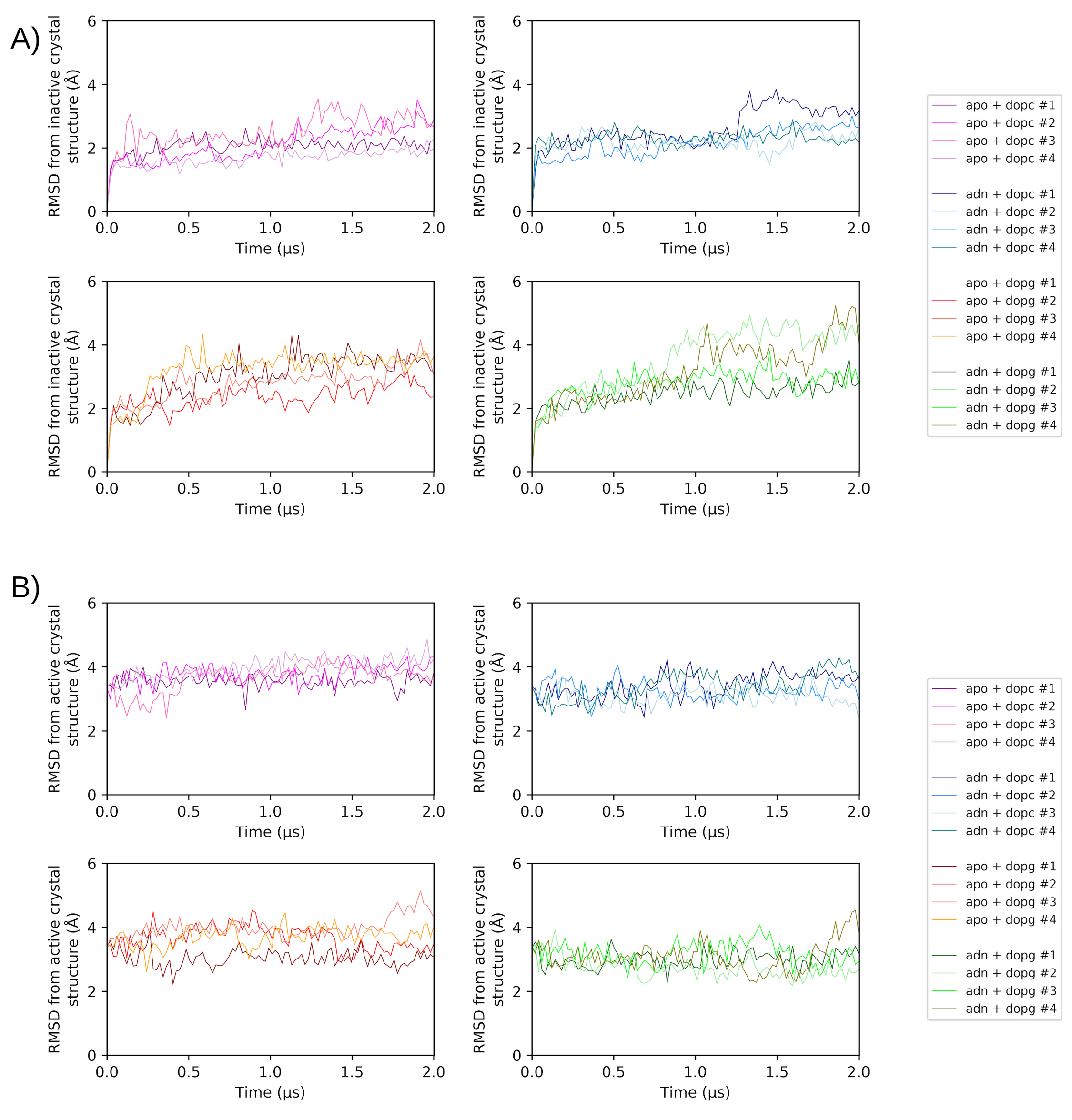

Supplement: S5 Fig — A) RMSD of helices 1–7 from the inactive crystal structure (PDB entry: 4EIY) and B) with respect to the active crystal structure of A2aR (PDB entry: 6GDG). MD simulations are performed in quadruplicate, with or without bound adenosine (ADN) in DOPC or DOPG homogeneous membranes. (TIF) [file pcbi.1007818.s006.tif]

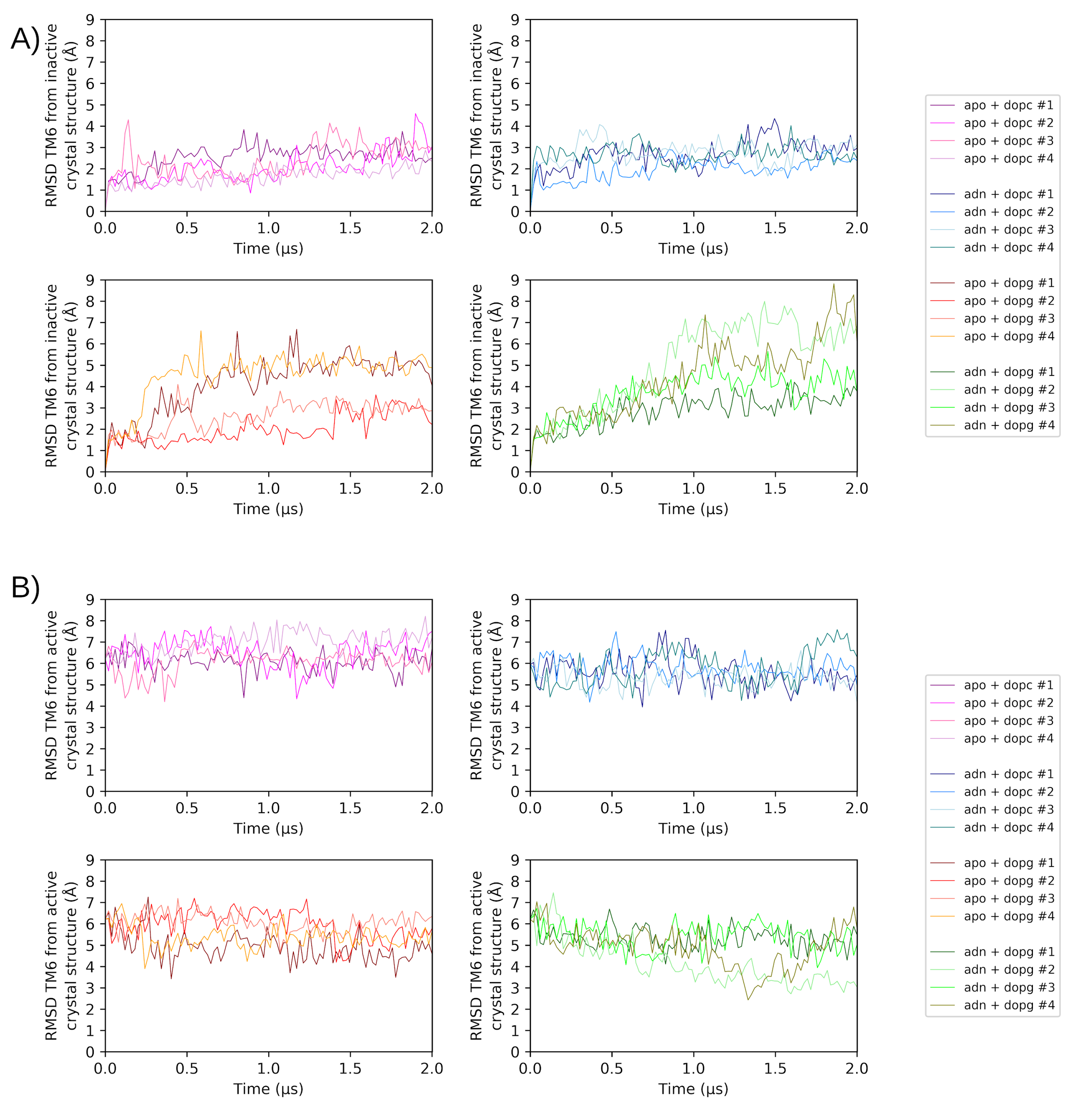

Supplement: S6 Fig — A) RMSD from the starting inactive A2aR crystal structure (PDB entry: 4EIY) and B) with respect to the active A2aR crystal structure (PDB entry: 6GDG). MD simulations are performed in quadruplicate, with or without bound adenosine (ADN) and in DOPC or DOPG homogeneous membranes. (TIF) [file pcbi.1007818.s007.tif]

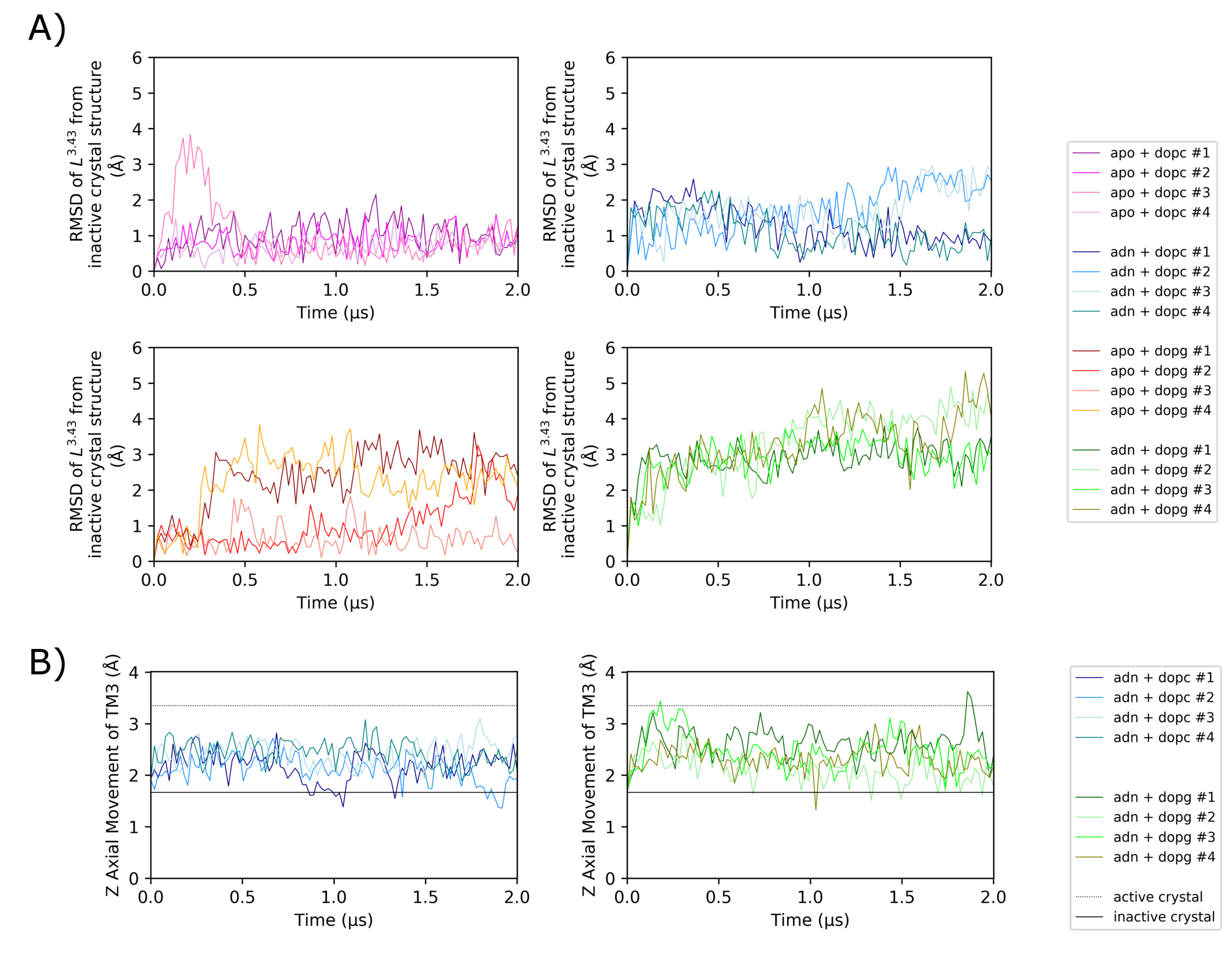

Supplement: S7 Fig — A) RMSD of residue L3.43 on TM3 compared to the inactive crystal structure (PDB entry: 4EIY) and B) assessment of vertical movement of TM3 along Z-axis. MD simulations are performed in quadruplicate with or without bound adenosine (ADN) and in DOPC or DOPG homogeneous membranes. (TIF) [file pcbi.1007818.s008.tif]

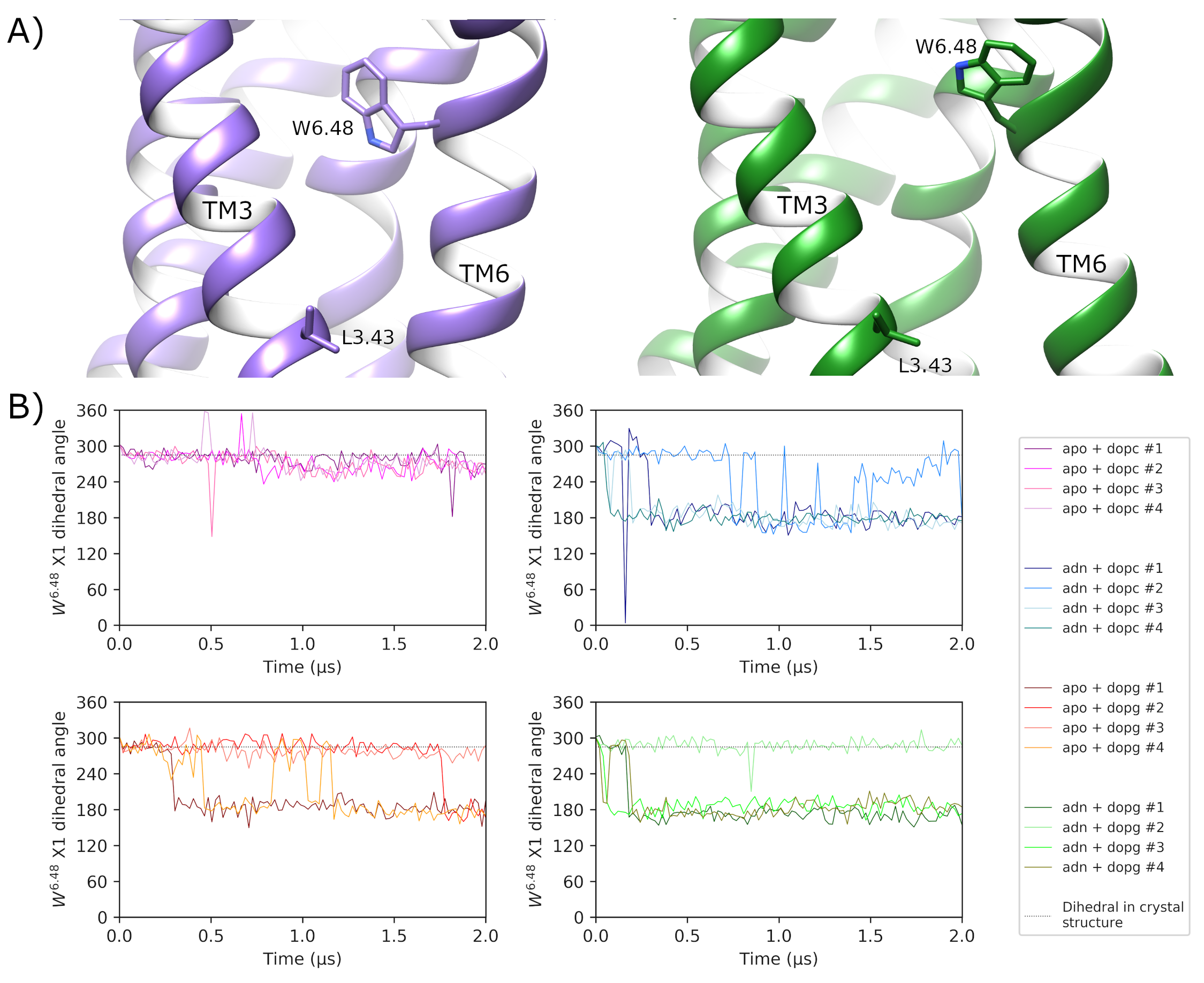

Supplement: S8 Fig — A) W2466.48 rotameric switch starting from gauche(-) (285°) (belonging to replica #2 from 1.7 μs in APO embedded in DOPC, in magenta) to trans (180°) during MD simulation replica #4 from 1.8 μs in DOPG with bound adenosine (in green). B) χ1 dihedral angle of residue W2466.48 over time. MD simulations are performed in quadruplicate, with or without bound adenosine (ADN) in DOPC or DOPG homogeneous membranes. (TIF) [file pcbi.1007818.s009.tif]

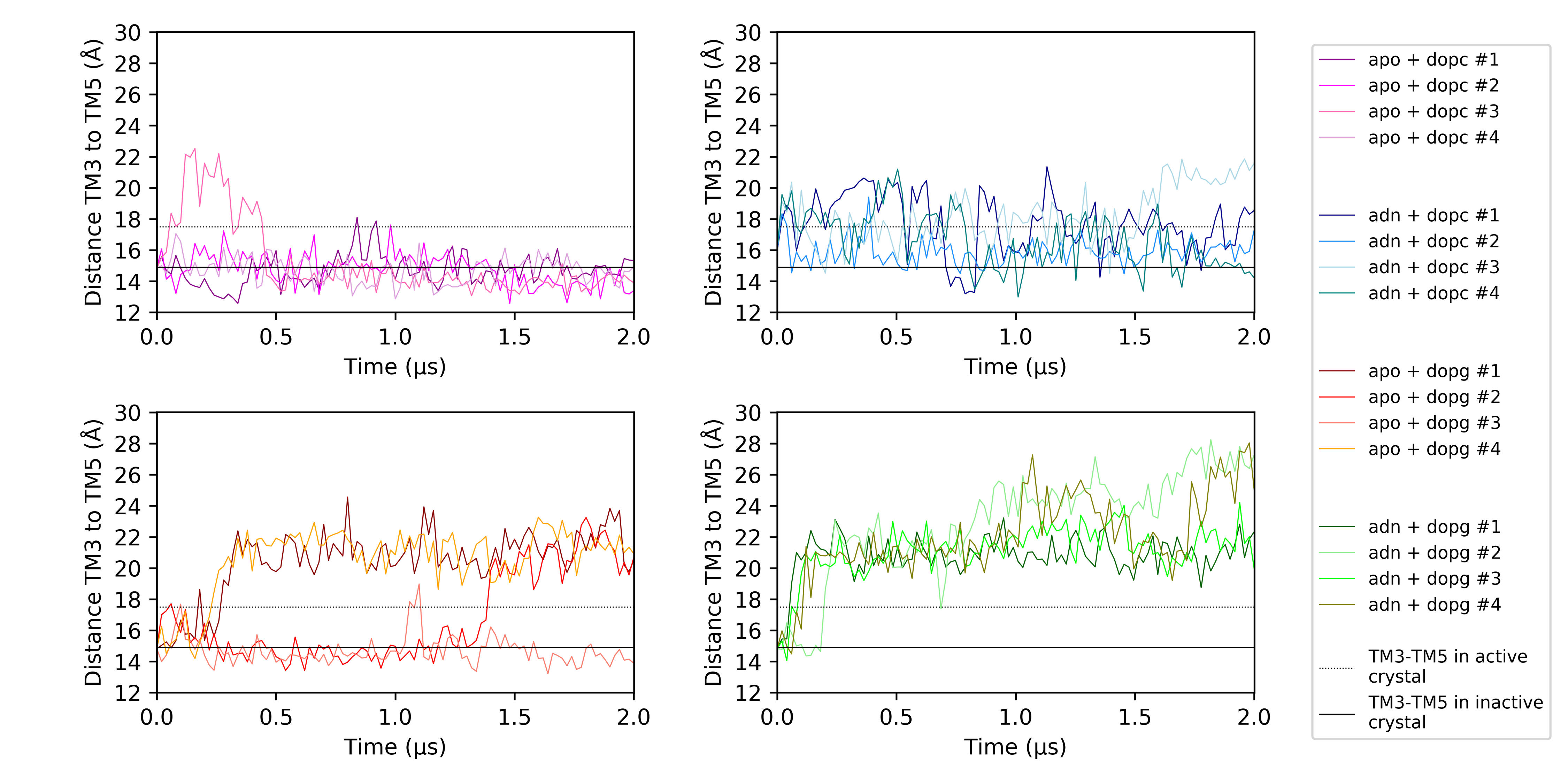

Supplement: S9 Fig — Distance between TM3-TM5 is measured between Cα atoms of R1023.50 and Q2075.68. MD simulations are performed in quadruplicate, with or without bound adenosine (ADN) in DOPC or DOPG homogeneous membranes. (TIF) [file pcbi.1007818.s010.tif]

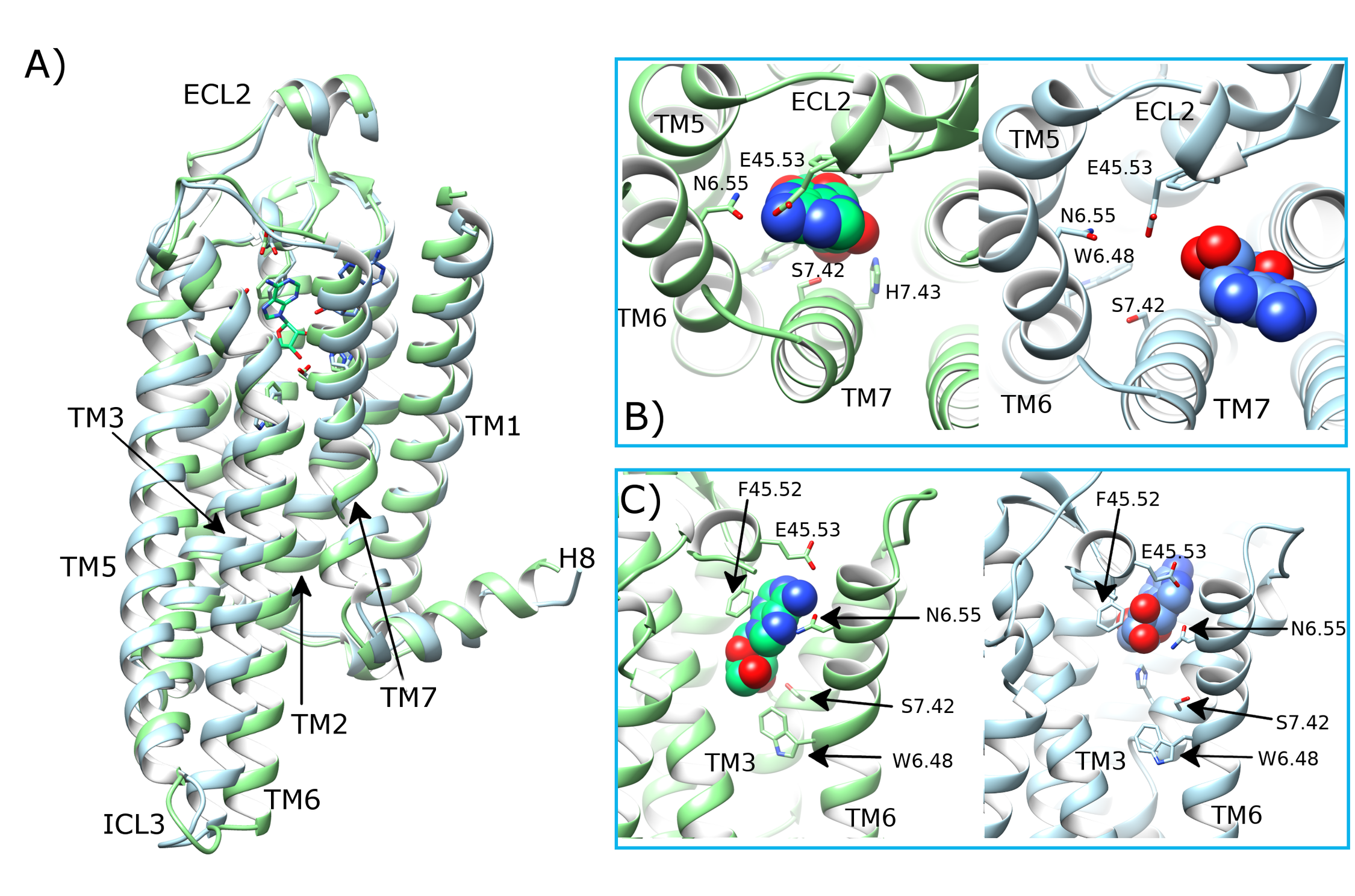

Supplement: S10 Fig — A) Superposition of the intermediate crystal structure of A2aR (PDB entry: 2YDO, light green) and an MD-generated conformation achieved within a DOPC membrane bound to adenosine (in blue, belonging to replica #1 at 1.9 μs) showing B) and C) ligand atoms as spheres and selected residues making protein-ligand interactions as sticks. Intracellular loop (ICL) 3, extracellular loop (ECL) 2, and transmembrane (TM) helices 1–3 and 5–7 are labelled. (TIF) [file pcbi.1007818.s011.tif]

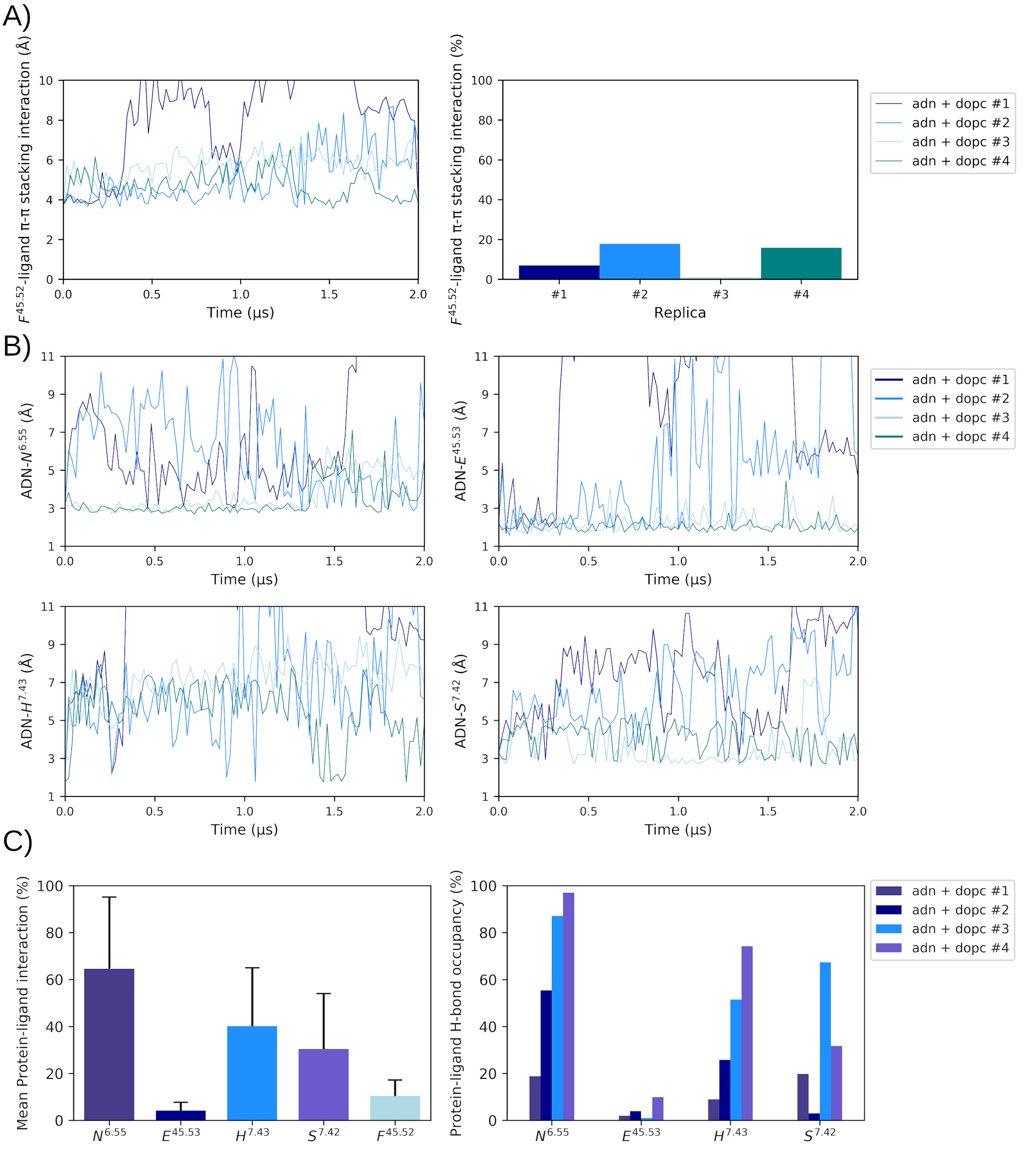

Supplement: S11 Fig — A) Left: distance of F45.52 with respect to ribose moiety of adenosine (ADN). Right: frequency (%) of protein-ligand π-π stacking (within range of 0.0 to 4.0 Å) over 2 μs. B) Evaluation of protein-ligand H-bond distances formed by residues: N2536.55, E16945.53, H2787.43, S2777.42 (N—O or O—O). C) Mean protein-ligand interactions (%) and protein-ligand H-bond occupancies per replica (%) for selected residues. (TIF) [file pcbi.1007818.s012.tif]

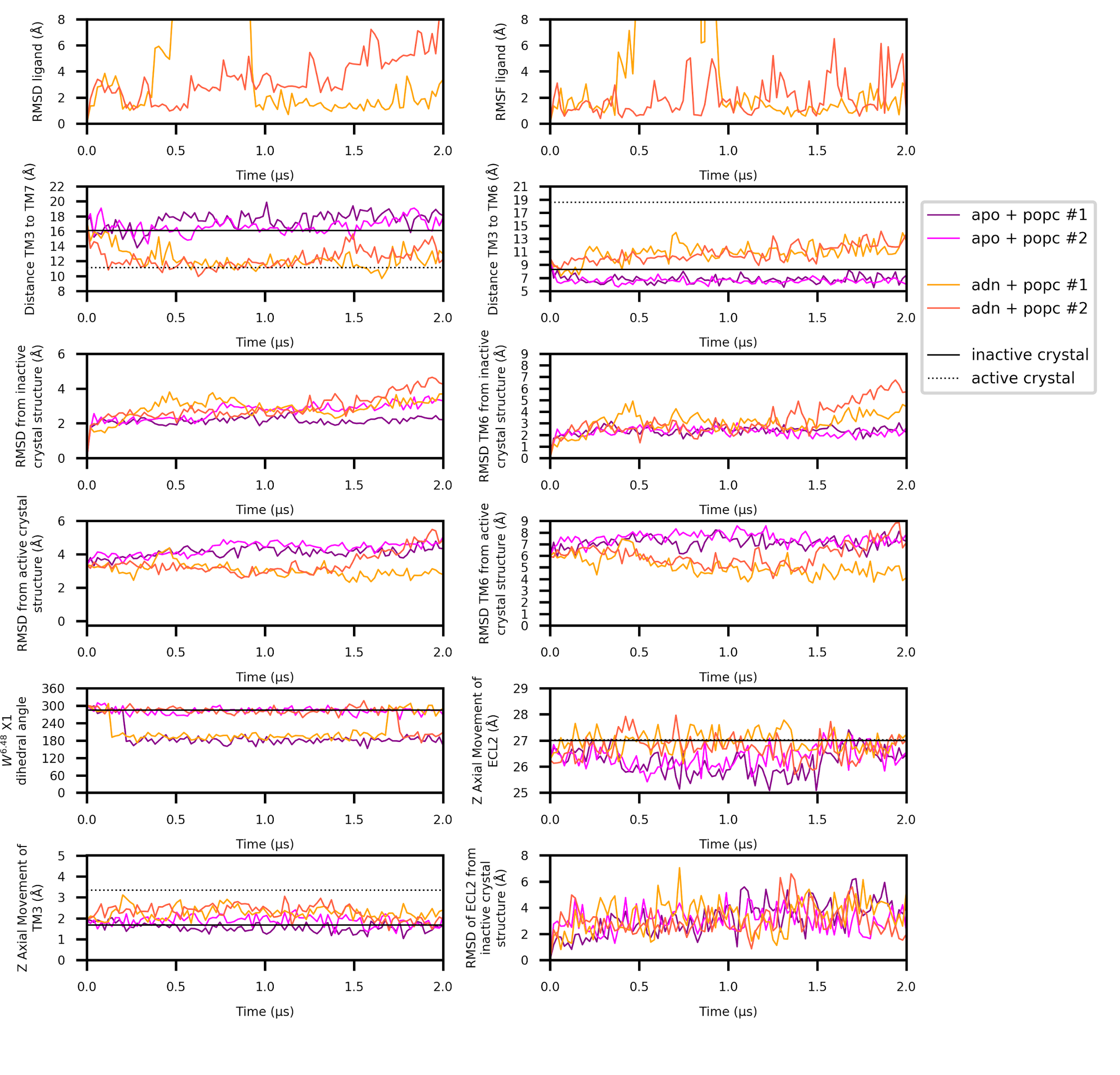

Supplement: S12 Fig — Top row: RMSD and conformational fluctuation (RMSF) of bound adenosine ligand; second row: TM3-TM7 and ionic lock (TM3-TM6) inter-helical distances; third row: RMSD of whole TMD (TMs 1–7) or only TM6; fourth row: RMSD compared to active crystal structure (PDB id: 6GDG) of whole TMD (TMs 1–7) or only TM6; fifth row: χ1 dihedral angle of W2466.48 on TM6 starting from gauche(-) crystal position (285°), and vertical movement of extracellular loop 2 (ECL2); bottom row: vertical movement of TM3 and RMSD of ECL2. MD simulations are performed in duplicate in POPC homogeneous membranes. (TIF) [file pcbi.1007818.s013.tif]

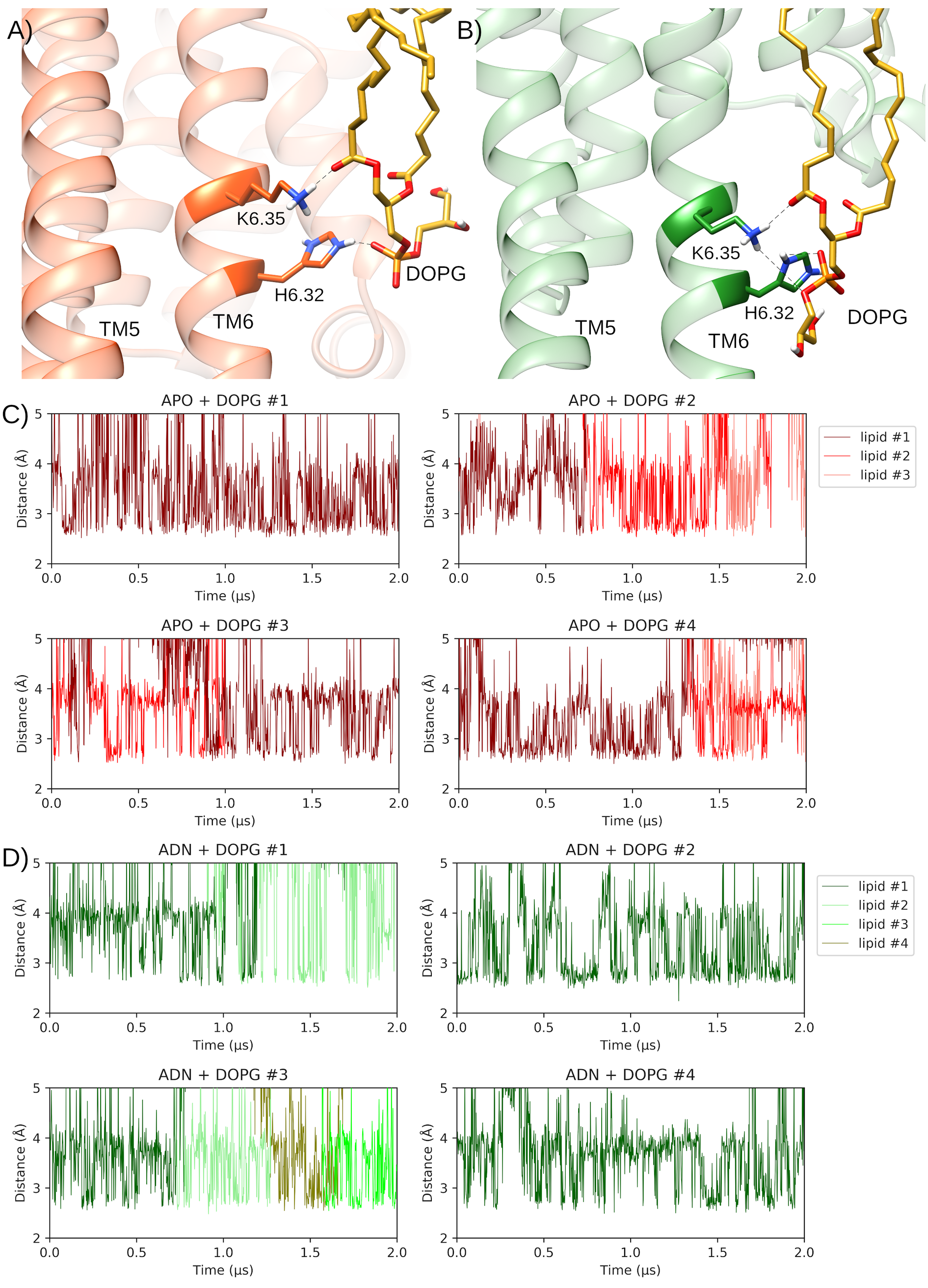

Supplement: S13 Fig — A) Residues H2306.32 and K2336.35 on TM6 of A2aR in apo state (orange) and B) A2aR with bound adenosine (green) interacting with DOPG lipid (gold). Histidine-lipid interaction distances over time in four replicas of A2aR in DOPG membrane in C) apo state and D) adenosine-bound (ADN), respectively. (TIF) [file pcbi.1007818.s014.tif]

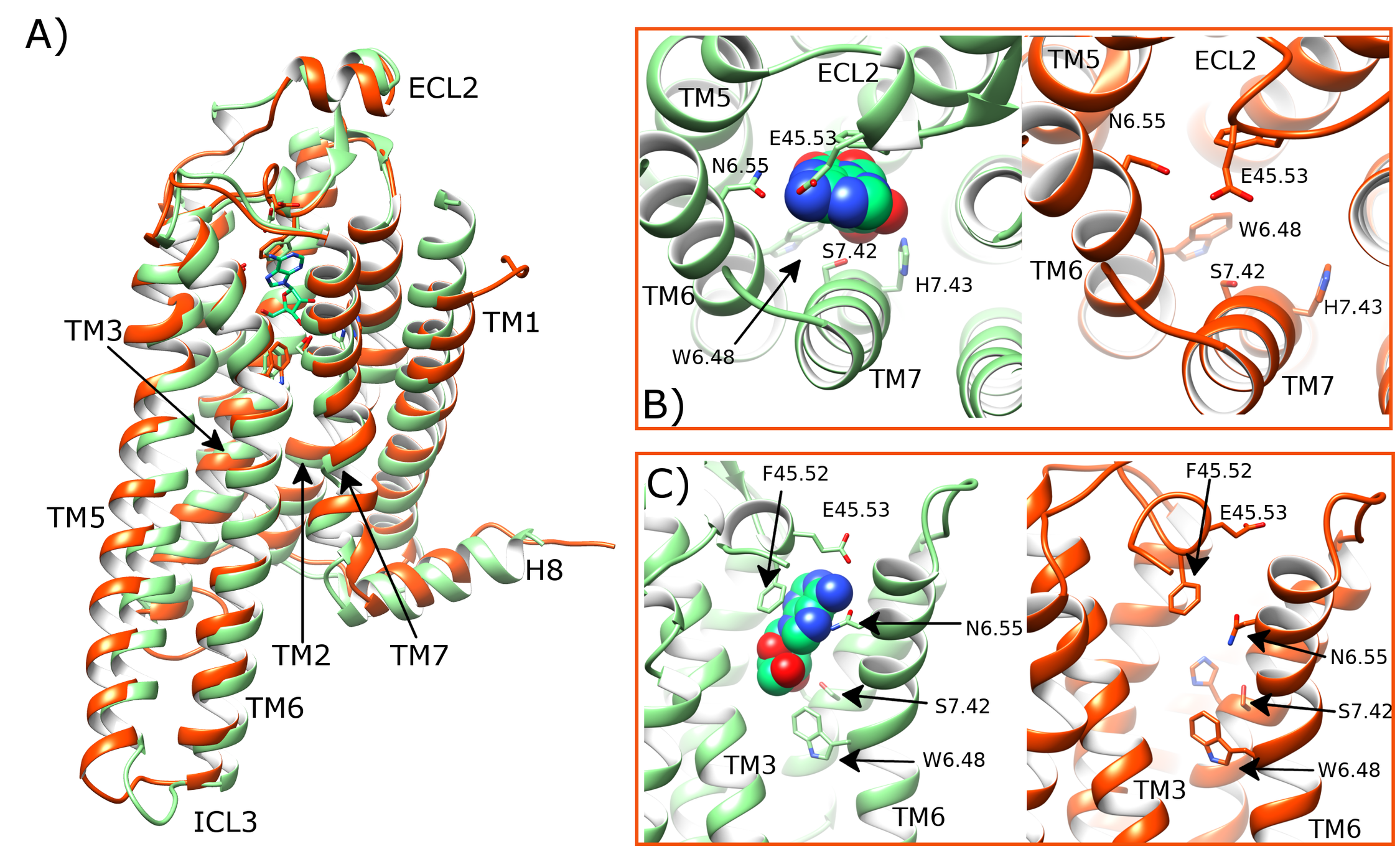

Supplement: S14 Fig — A) Comparison of the intermediate crystal structure of A2aR (PDB entry: 2YDO, light green) and an MD-generated apo conformation achieved within a DOPG membrane (in red, belonging to replica #2 at 1.6 μs) showing B) and C) selected residues delineating the orthosteric pocket. Intracellular loop (ICL) 3, extracellular loop (ECL) 2, and transmembrane (TM) helices 1–3, 5–7 are labelled. (TIF) [file pcbi.1007818.s015.tif]

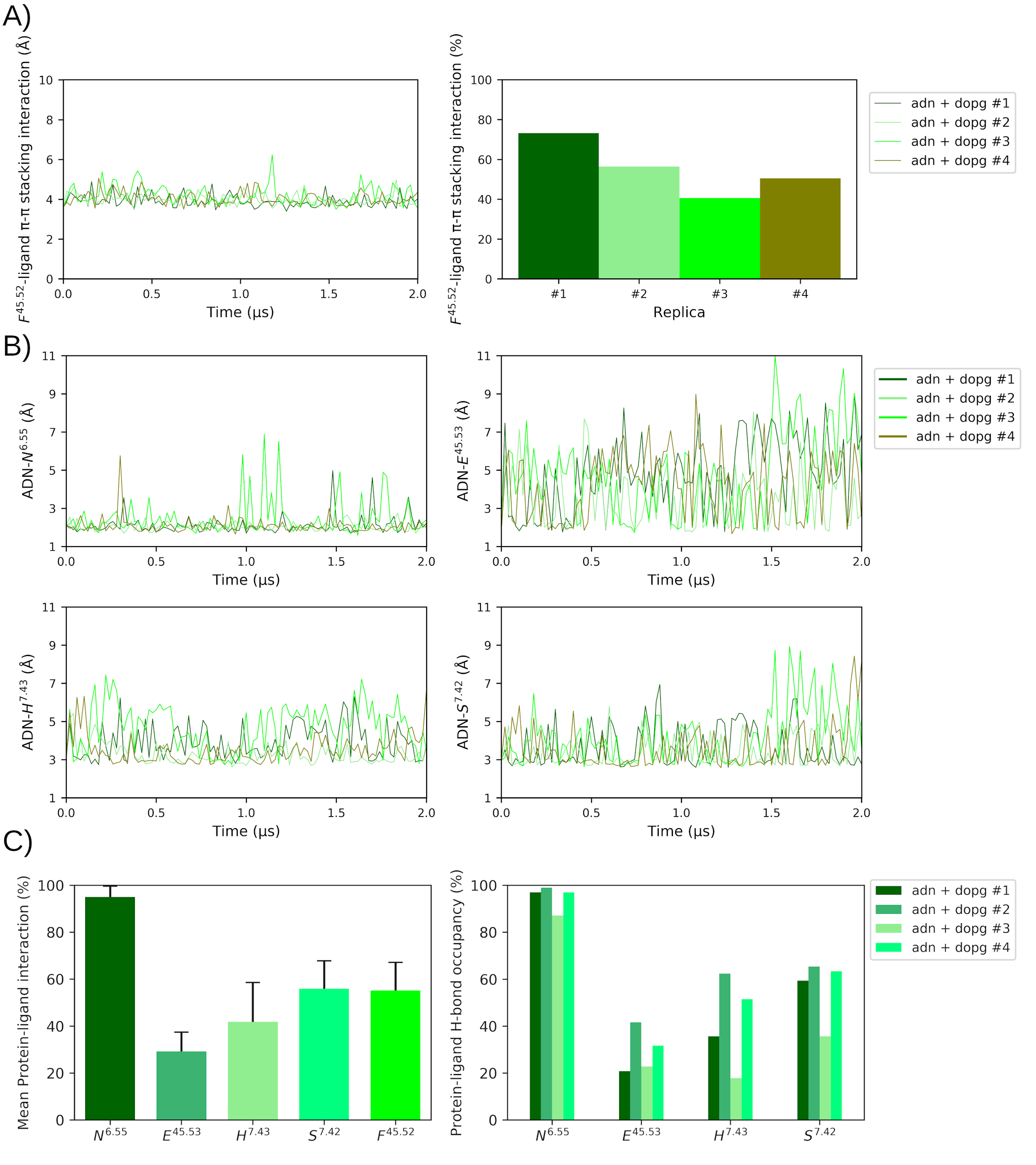

Supplement: S15 Fig — A) Left: distance of F45.52 with respect to ribose moiety of adenosine (ADN). Right: frequency (%) of protein-ligand π-π stacking (within range of 0.0 to 4.0 Å) over 2 μs. B) Evaluation of protein-ligand H-bond distances formed by residues: N2536.55, E16945.53, H2787.43, S2777.42 (N—O or O—O). C) Mean protein-ligand interactions (%) and protein-ligand H-bond occupancies per replica (%) for selected residues. (TIF) [file pcbi.1007818.s016.tif]

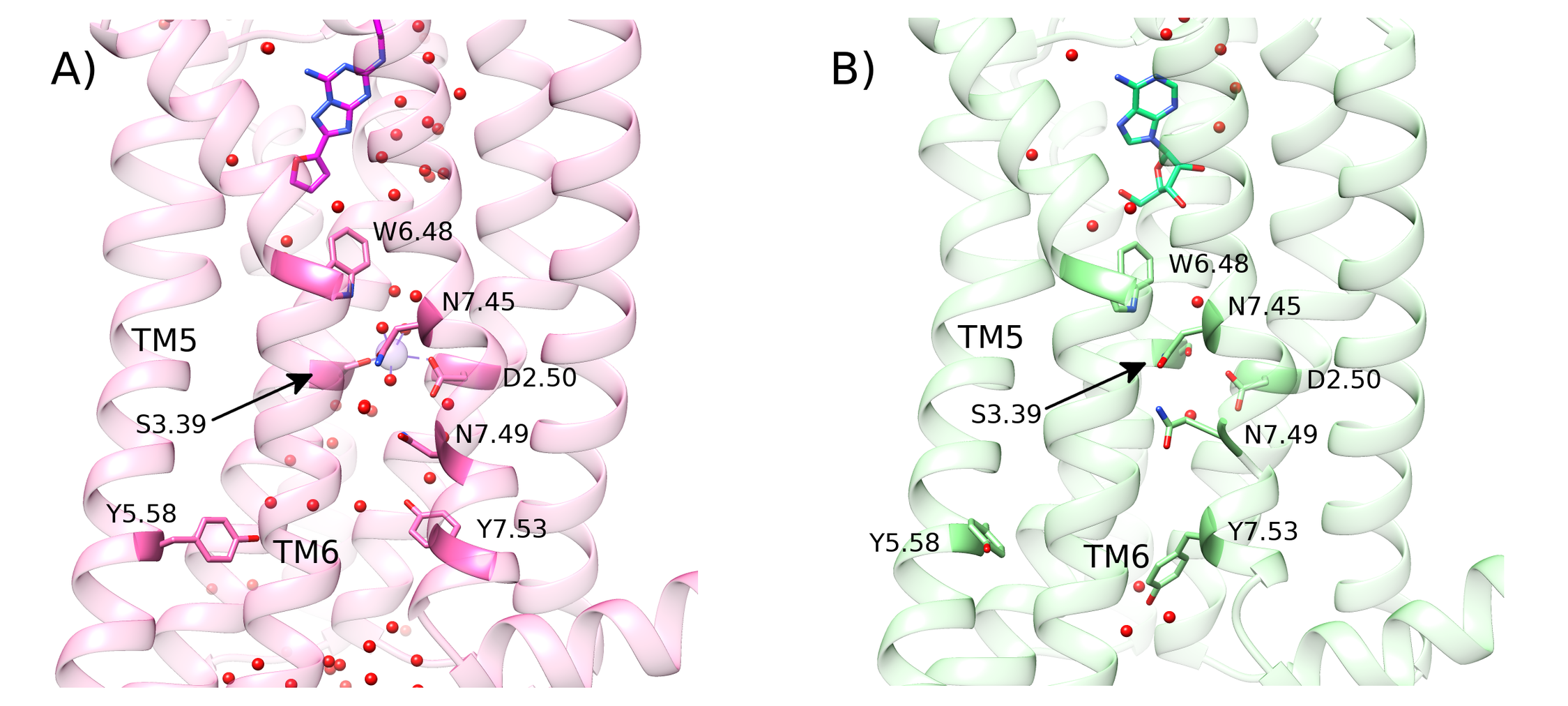

Supplement: S16 Fig — The water network retrieved from: A) the inactive crystal structure (PDB entry: 4EIY, pink) and B) the intermediate crystal structure (PDB entry: 2YDO, light green). Residues and ligands are shown as sticks and water molecules are shown as red spheres. (TIF) [file pcbi.1007818.s017.tif]

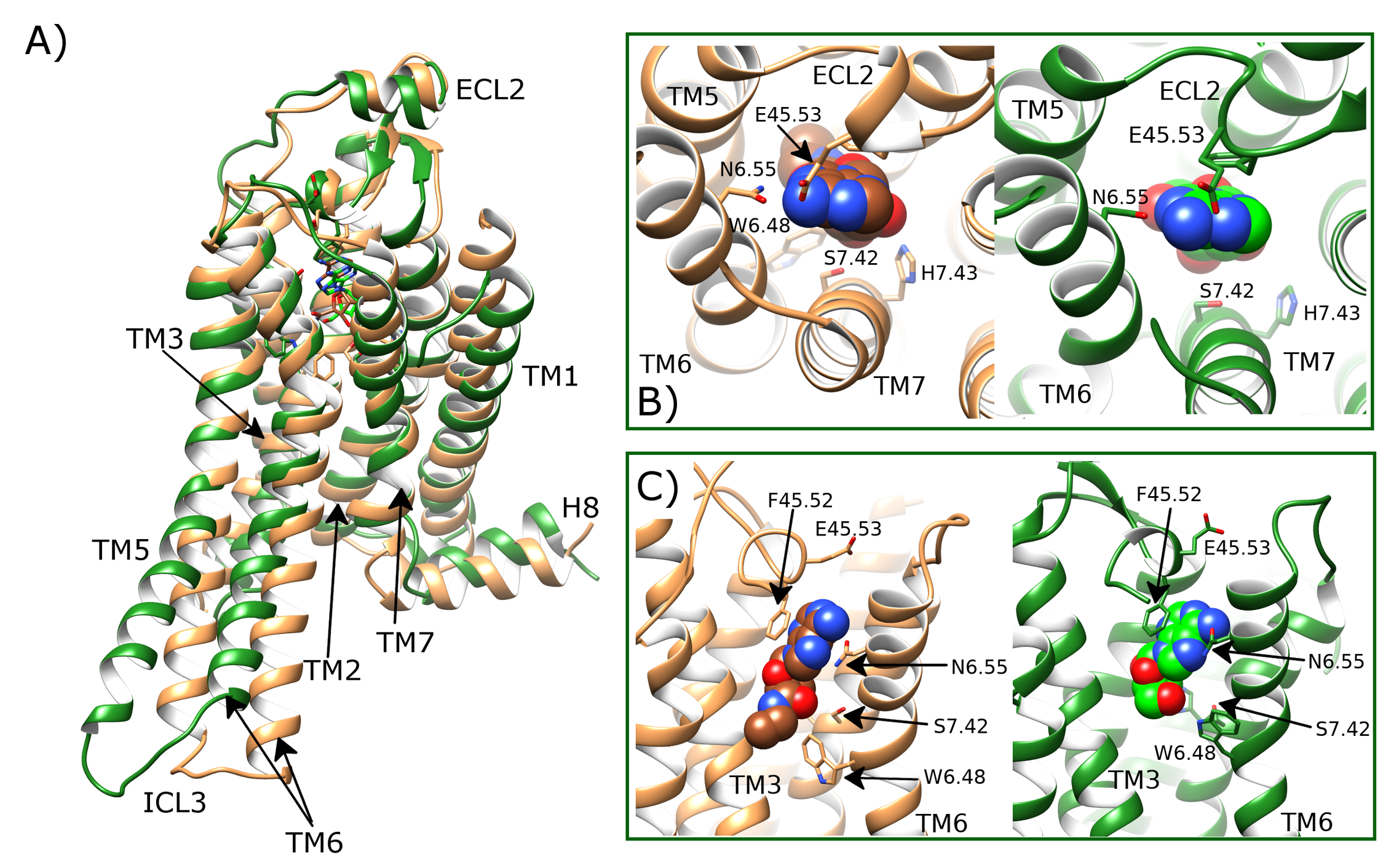

Supplement: S17 Fig — A) Comparison of an MD-generated conformation of A2aR bound to adenosine within a DOPG membrane (in green, belonging to replica #4 at 1.8 μs) with the active crystal structure of A2aR (brown, PDB entry: 6GDG) showing B) and C) ligand atoms as spheres and residues making protein-ligand interactions as sticks. Intracellular loop (ICL) 3, extracellular loop (ECL) 2, and transmembrane (TM) helices 1–3, 5–7 are labelled. (TIF) [file pcbi.1007818.s018.tif]

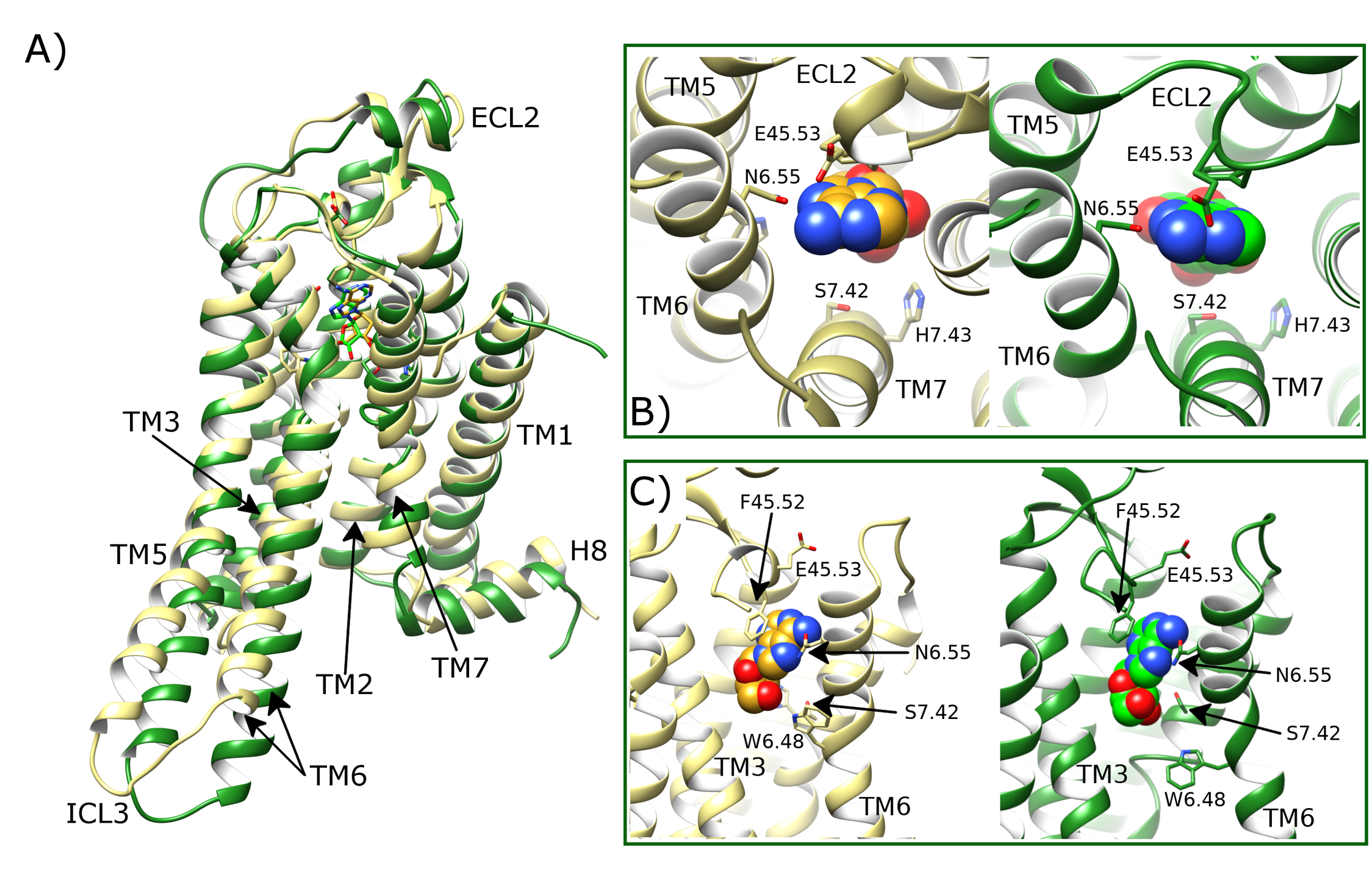

Supplement: S18 Fig — A) Comparison of an MD-generated conformation of A2aR bound to adenosine within DOPG belonging to replica #2 (at 1.6 μs, green) with respect to replica #4 (at 1.8 μs, olive green) showing B) and C) ligand atoms as spheres and residues making protein-ligand interactions as sticks. Intracellular loop (ICL) 3, extracellular loop (ECL) 2, and transmembrane (TM) helices 1–3, 5–7 are labelled. (TIF) [file pcbi.1007818.s019.tif]

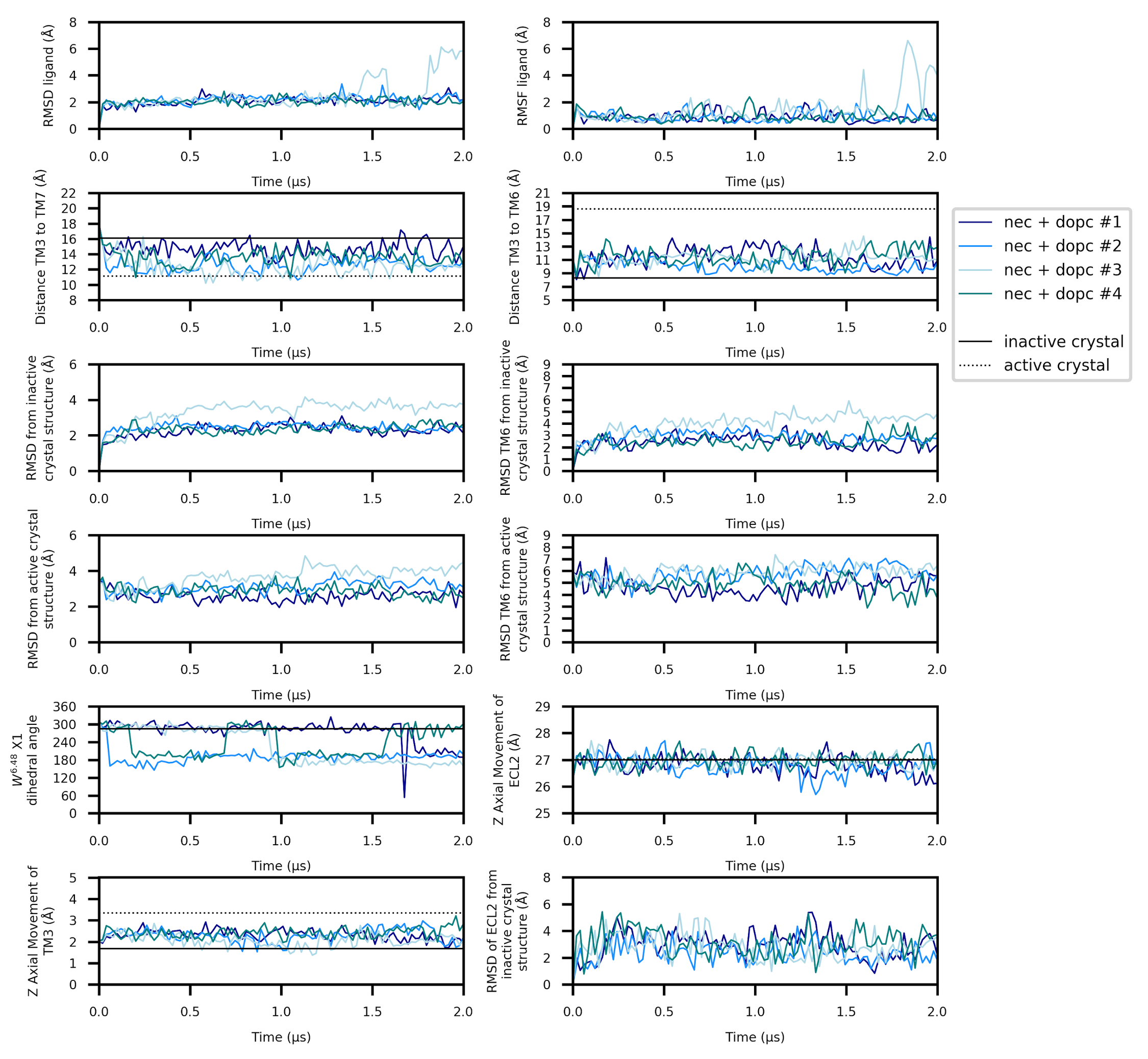

Supplement: S19 Fig — Top row: RMSD and conformational fluctuation (RMSF) of bound NECA ligand; second row: TM3-TM7 and ionic lock (TM3-TM6) inter-helical distances; third row: RMSD of whole TMD (TMs 1–7) or only TM6; fourth row: RMSD compared to active crystal structure (PDB id: 6GDG) of whole TMD (TMs 1–7) or only TM6; fifth row: χ1 dihedral angle of W2466.48 on TM6 starting from gauche(-) crystal position (285°), and vertical movement of extracellular loop 2 (ECL2); bottom row: vertical movement of TM3 and RMSD of ECL2. MD simulations are performed in quadruplicate in DOPC homogeneous membranes. (TIF) [file pcbi.1007818.s020.tif]

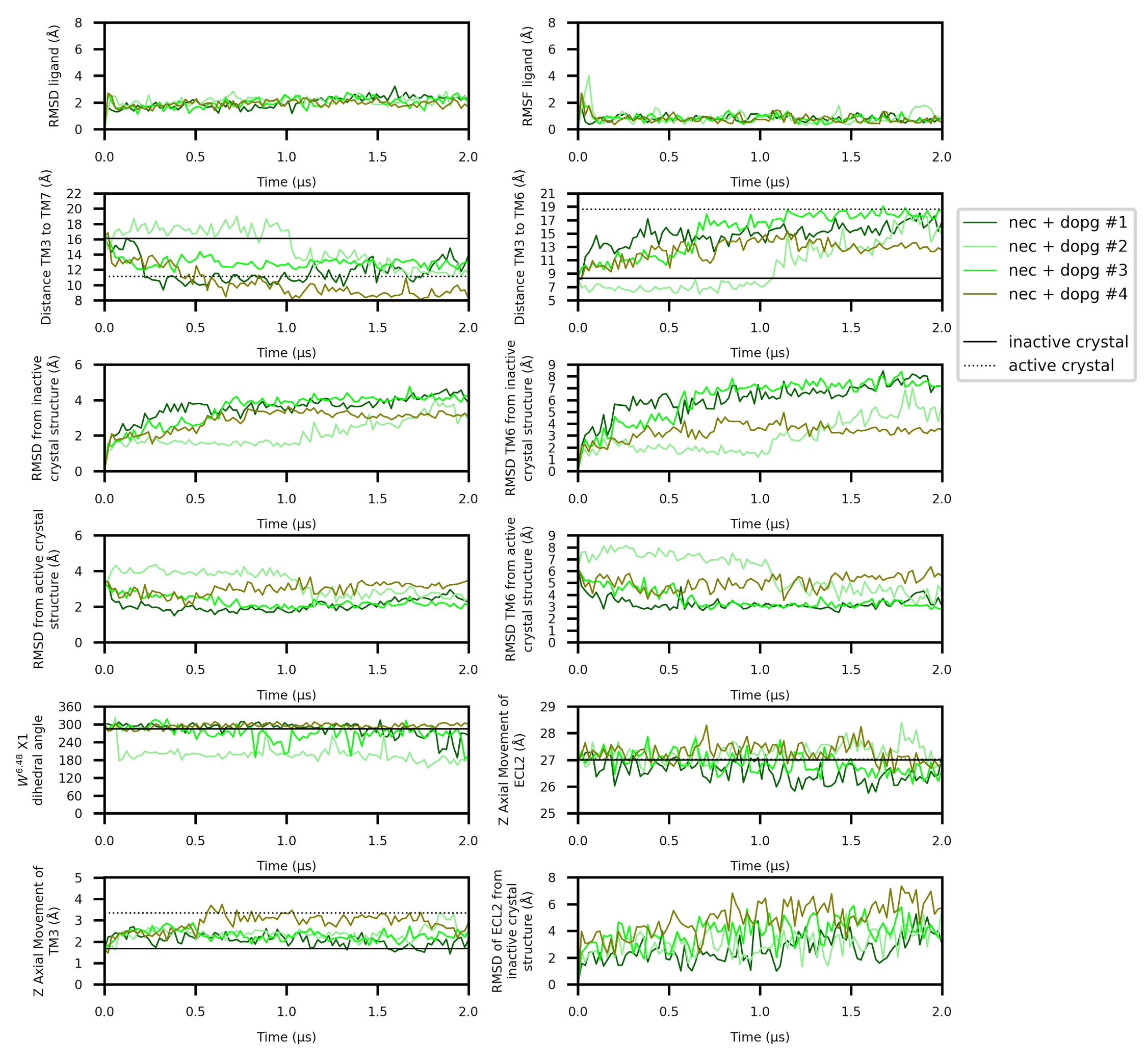

Supplement: S20 Fig — Top row: RMSD and conformational fluctuation (RMSF) of bound NECA ligand; second row: TM3-TM7 and ionic lock (TM3-TM6) inter-helical distances; third row: RMSD of whole TMD (TMs 1–7) or only TM6; fourth row: RMSD compared to active crystal structure (PDB id: 6GDG) of whole TMD (TMs 1–7) or only TM6; fifth row: χ1 dihedral angle of W2466.48 on TM6 starting from gauche(-) crystal position (285°), and vertical movement of extracellular loop 2 (ECL2); bottom row: vertical movement of TM3 and RMSD of ECL2. MD simulations are performed in quadruplicate in DOPG homogeneous membranes. (TIF) [file pcbi.1007818.s021.tif]

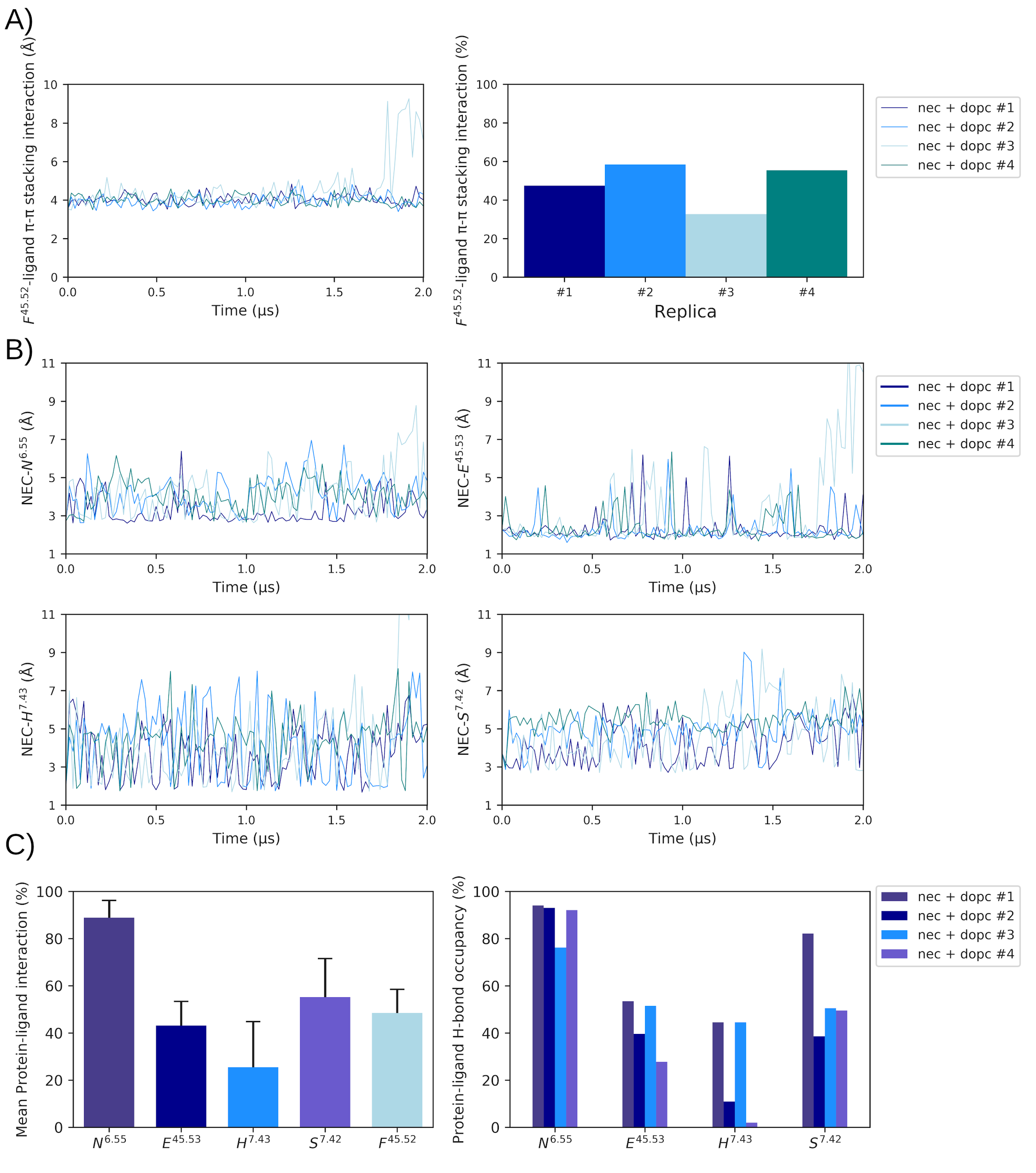

Supplement: S21 Fig — A) Left: distance of F45.52 with respect to ribose moiety of NECA. Right: frequency (%) of protein-ligand π-π stacking (within range of 0.0 to 4.0 Å) over 2 μs. B) Evaluation of protein-ligand H-bond distances formed by residues: N2536.55, E16945.53, H2787.43, S2777.42 (N—O or O—O). C) Mean protein-ligand interactions (%) and protein-ligand H-bond occupancies per replica (%) for selected residues. (TIF) [file pcbi.1007818.s022.tif]

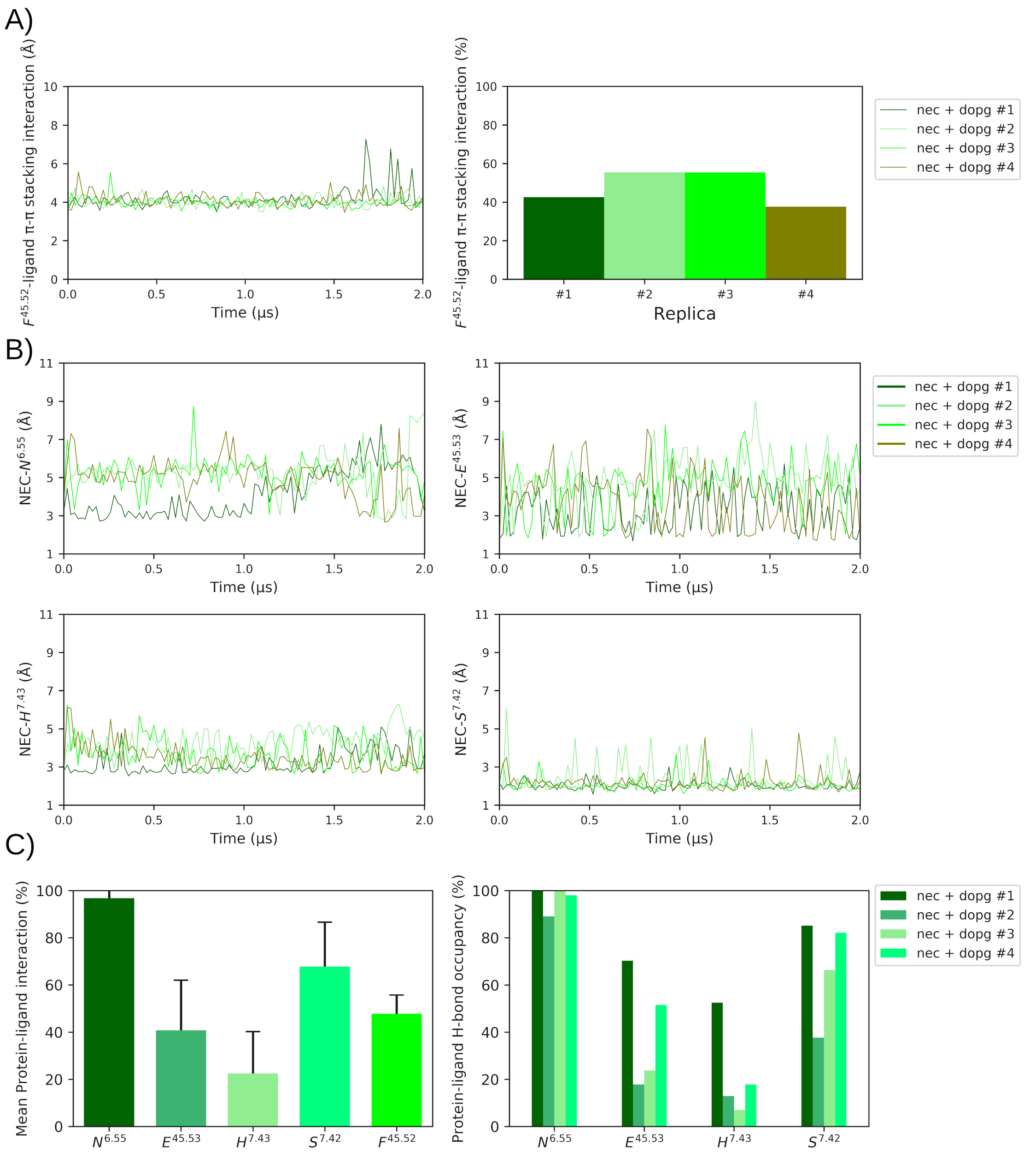

Supplement: S22 Fig — A) Left: Distance of F45.52 with respect to ribose moiety of NECA. Right: frequency (%) of protein-ligand π-π stacking (within range of 0.0 to 4.0 Å) interaction over 2 μs. B) Evaluation of protein-ligand H-bond distances formed by residues: N2536.55, E16945.53, H2787.43, S2777.42 (N—O or O—O). C) Mean protein-ligand interactions (%) and protein-ligand H-bond occupancies per replica (%) for selected residues. (TIF) [file pcbi.1007818.s023.tif]

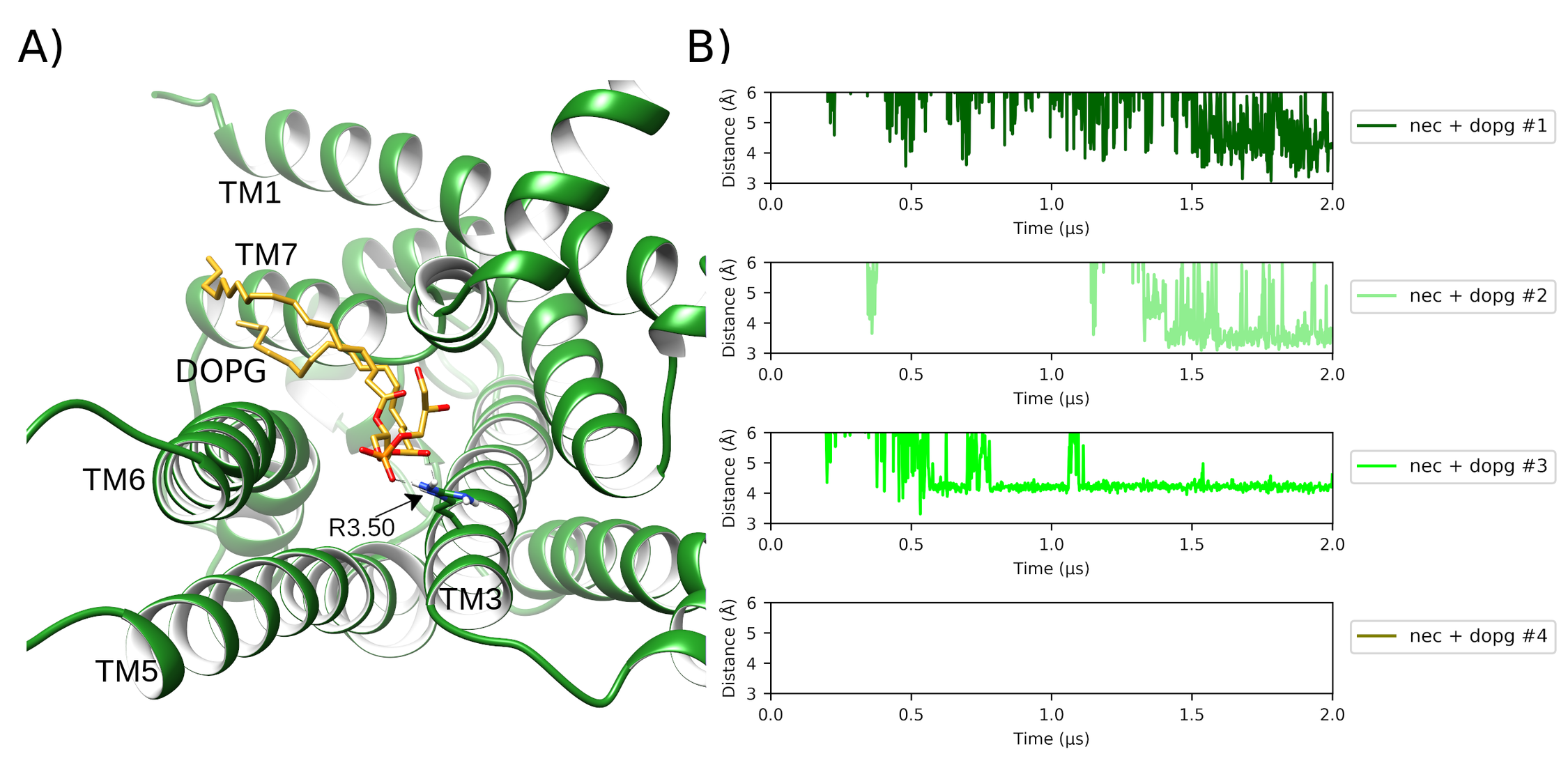

Supplement: S23 Fig — A) Electrostatic interaction between ionic-lock residue R1023.50 of A2aR (green) from an intracellular viewpoint and a DOPG lipid, which intrudes between TM6 and TM7 (snapshot belonging to replica #1 at 1.7 μs). B) Protein-lipid interaction distance over time between R1023.50 sidechain and lipid phosphate group in four replicas of NECA-bound A2aR in DOPG membrane. (TIF) [file pcbi.1007818.s024.tif]

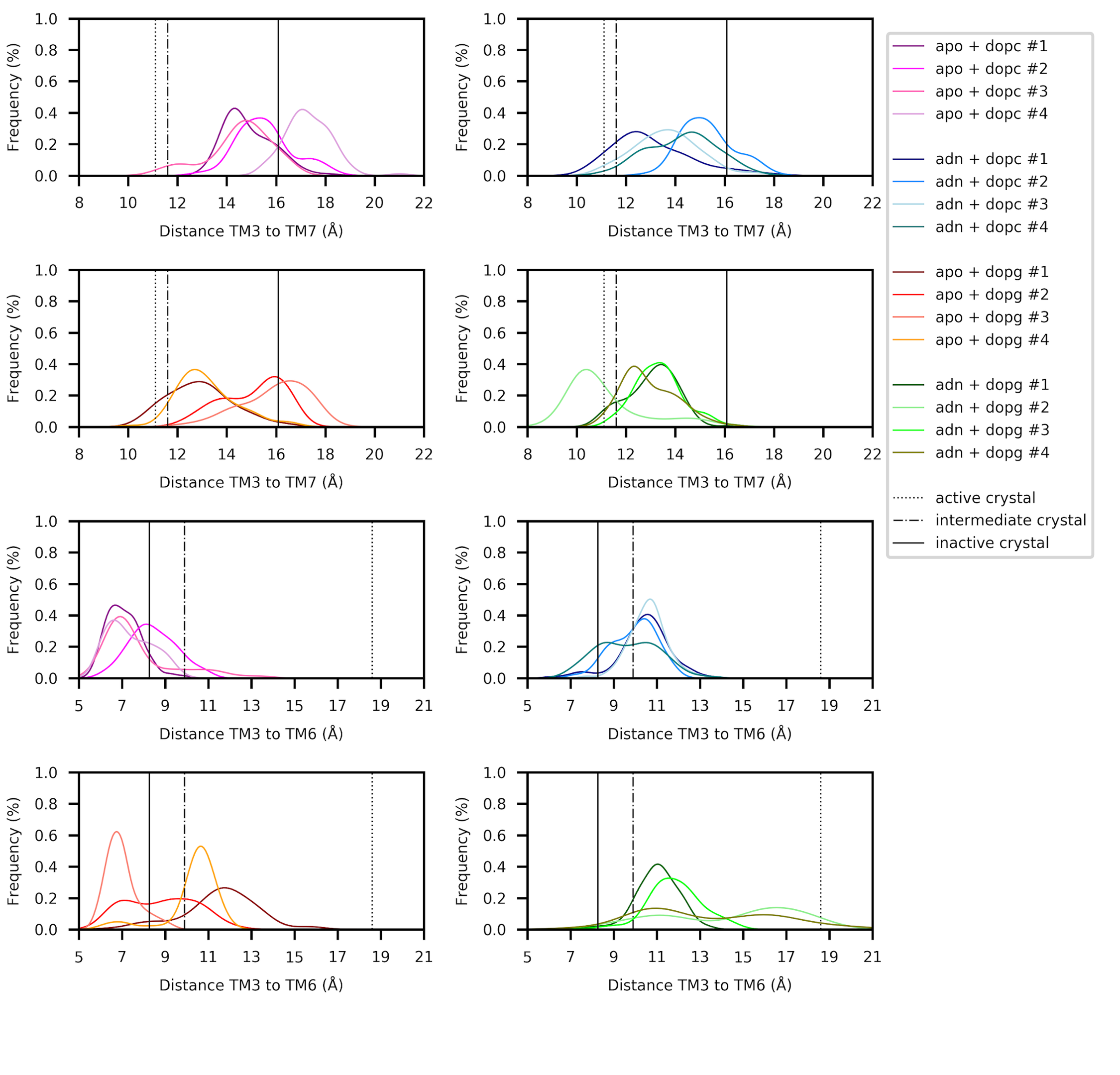

Supplement: S24 Fig — A) Population of receptor conformations according to distance between residues R3.50 and Y7.53 (TM3-TM7), and (B) between ionic lock residues R3.50 and E6.30 (TM3-TM6). Vertical black lines indicate values of inactive (PDB entry: 4EIY), intermediate (PDB entry: 2YDO) and active (PDB entry: 6GDG) crystal structures. (TIF) [file pcbi.1007818.s025.tif]

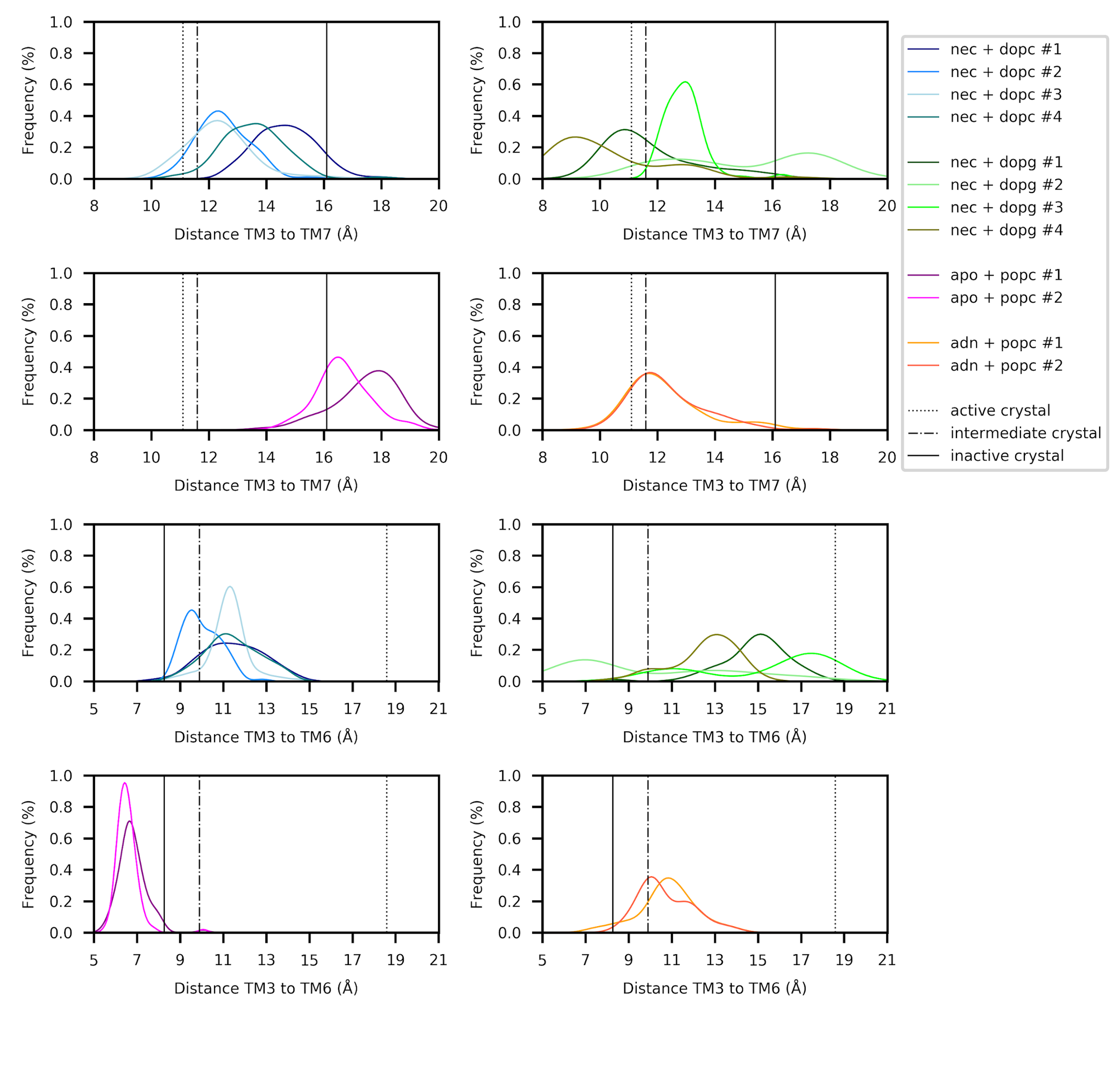

Supplement: S25 Fig — First and second rows: population of receptor conformations according to distance between R3.50 and Y7.53 (TM3-TM7); third and fourth rows: according to distance between ionic lock residues R3.50 and E6.30 (TM3-TM6). Vertical black lines indicate values of inactive (PDB entry: 4EIY), intermediate (PDB entry: 2YDO) and active (PDB entry: 6GDG) crystal structures. (TIF) [file pcbi.1007818.s026.tif]

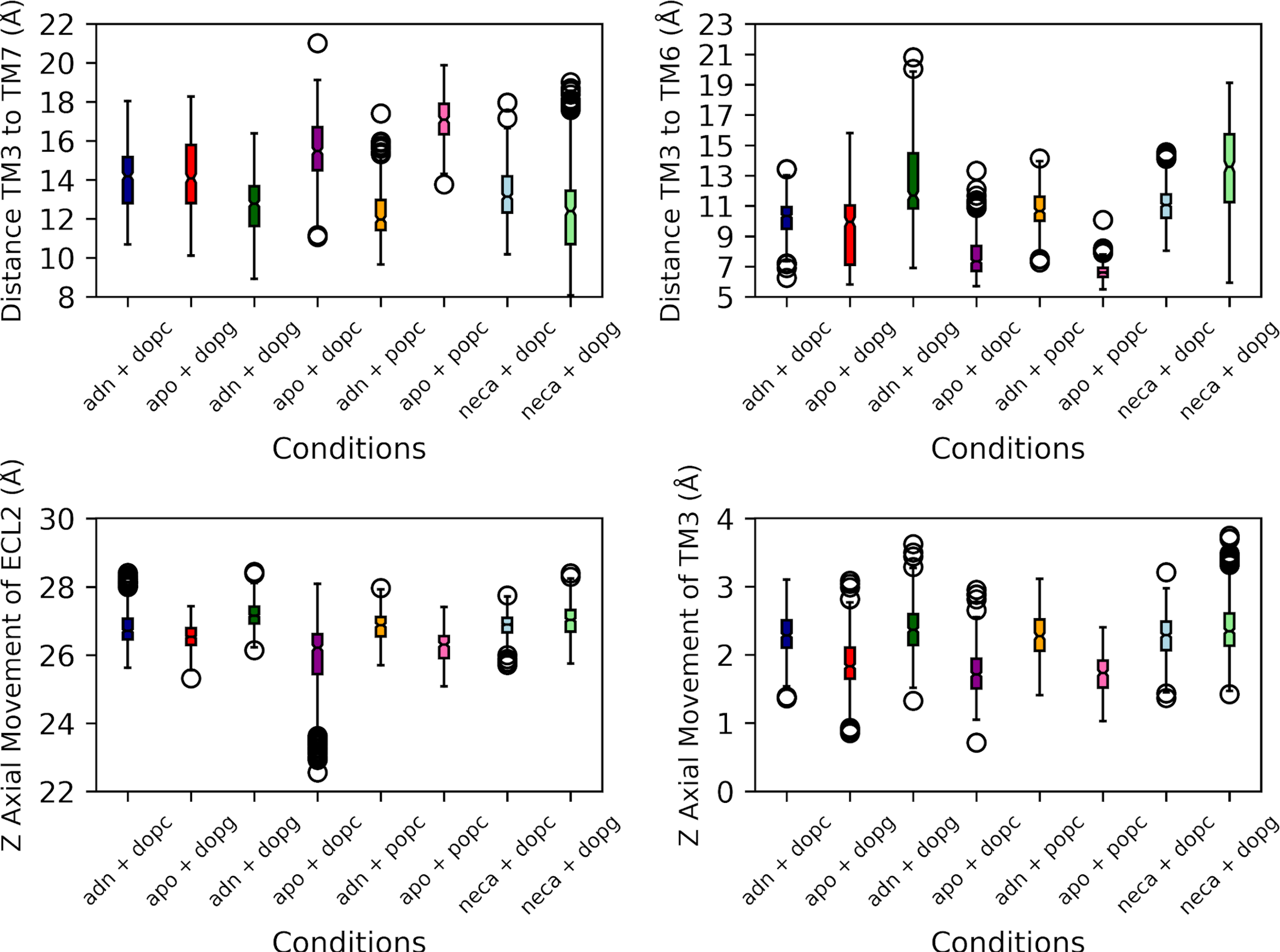

Supplement: S26 Fig — Top row: inter-helical distances between: residues R3.50 and Y7.53 (TM3-TM7), and ionic lock residues R3.50 and E6.30 (TM3-TM6); bottom row: vertical movements of extracellular loop 2 (ECL2) and TM3, respectively. MD simulations in DOPG or DOPC were performed in quadruplicate. MD simulations in POPC were performed in duplicate. (TIF) [file pcbi.1007818.s027.tif]

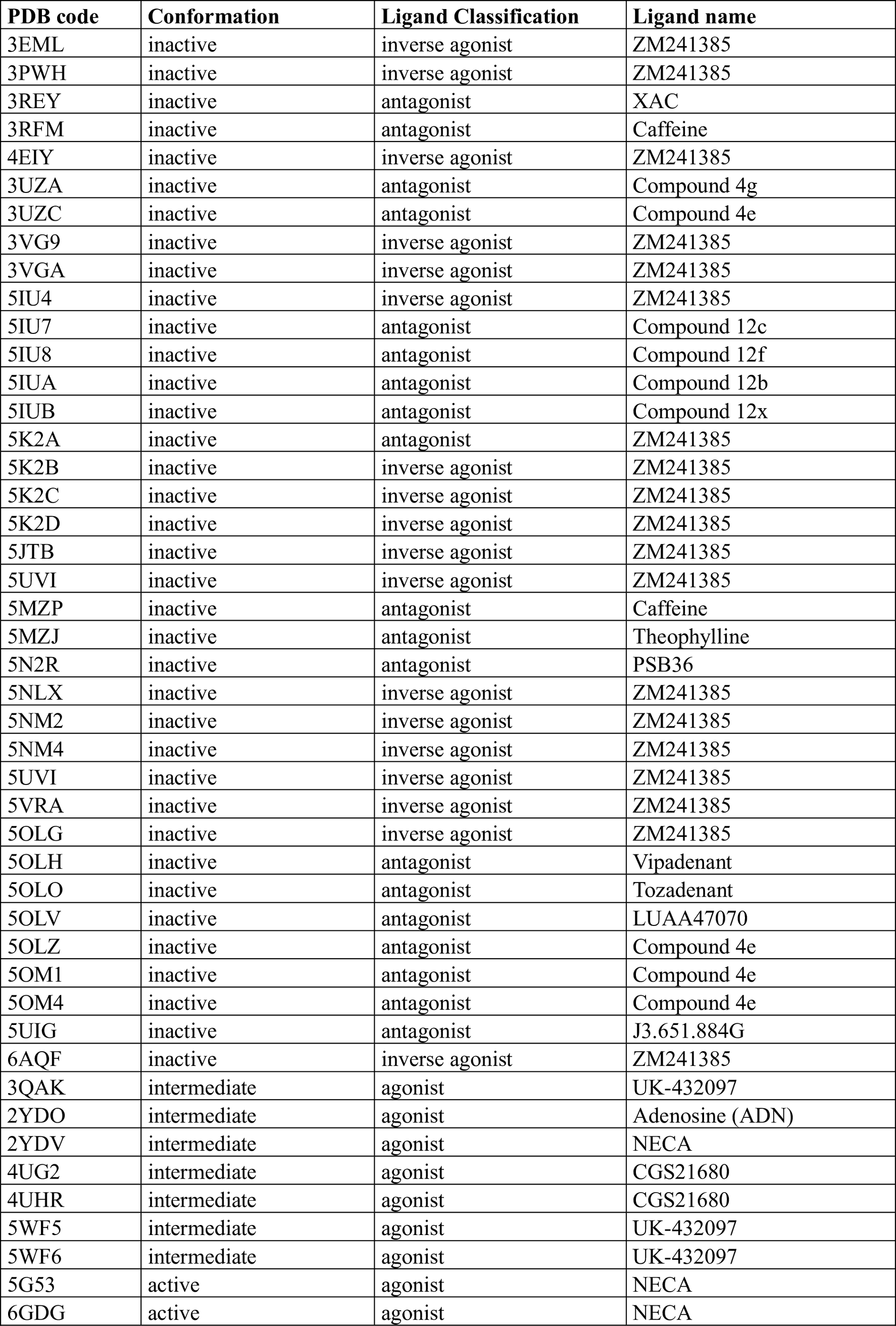

Supplement: S1 Table — (TIF) [file pcbi.1007818.s028.tif]

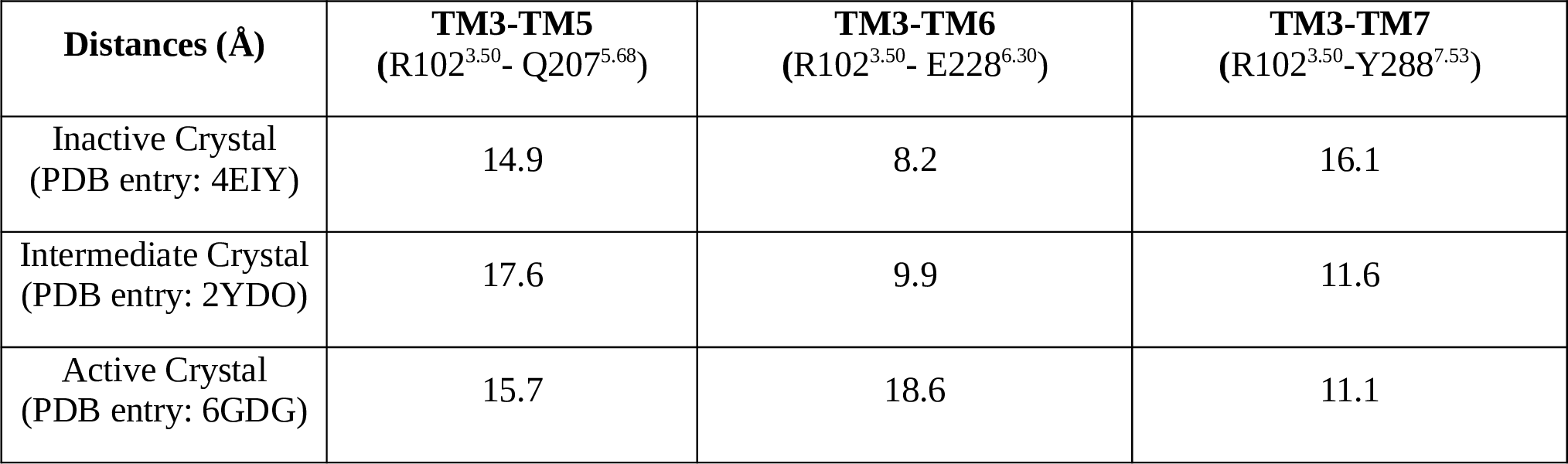

Supplement: S2 Table — Comparison of TM3-TM6, TM3-TM5 and TM3-TM7 inter-helical distances in active, intermediate and inactive crystal states. (TIF) [file pcbi.1007818.s029.tif]

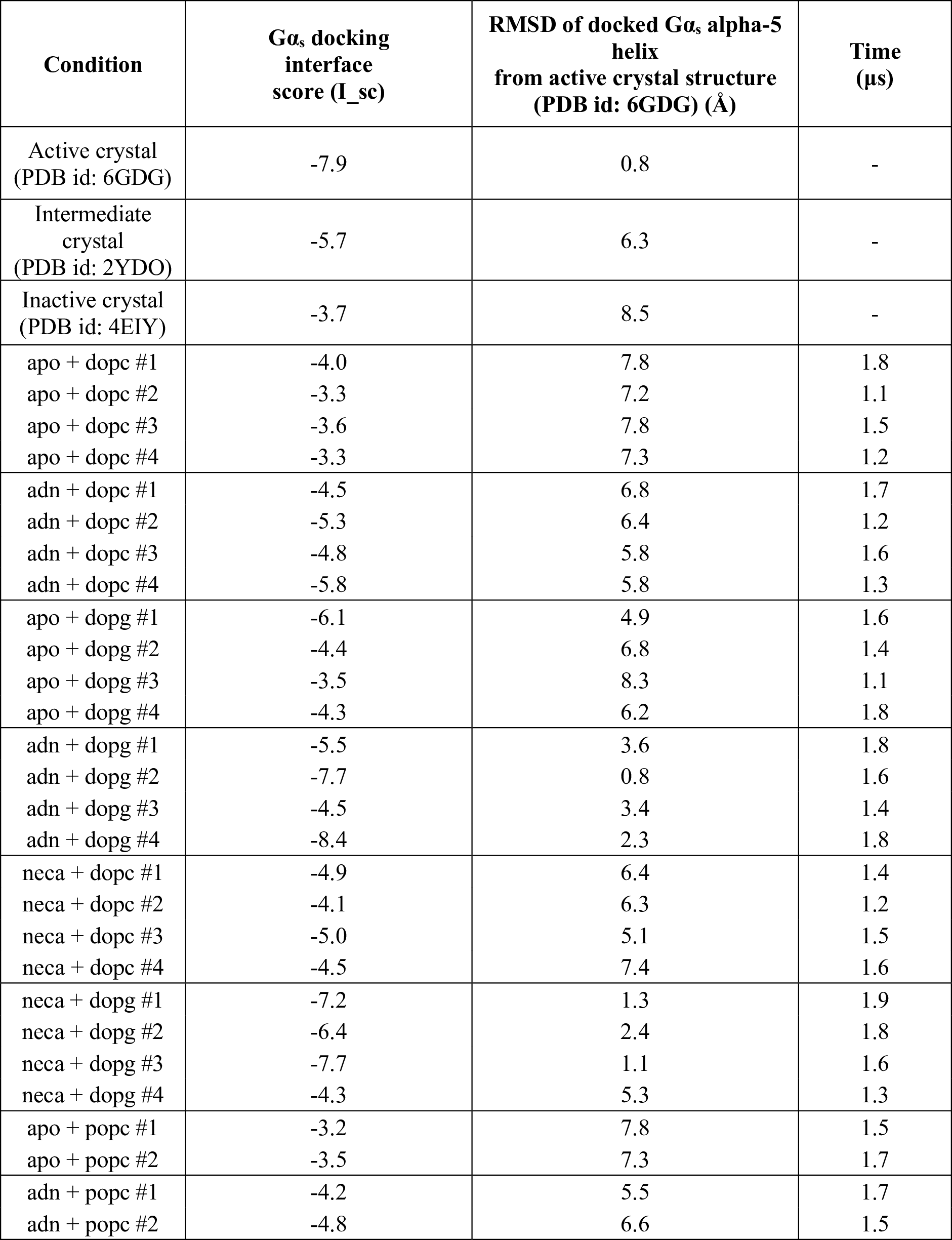

Supplement: S3 Table — Comparison of best docking quality of Gαs protein into inactive, intermediate and active crystals structures, and different MD-generated conformations of A2aR achieved under different conditions and performed in quadruplicate. (TIF) [file pcbi.1007818.s030.tif]
