# Supplementary material for: Insights into adenosine A2A receptor activation through cooperative modulation of agonist and allosteric lipid interactions
Source: PLoS Comput Biol. 2020 Apr 16;16(4):e1007818. doi: 10.1371/journal.pcbi.1007818 (PMC7188303; doi:10.1371/journal.pcbi.1007818)
Supplement: Attachment — Submitted filename: Bruzzese et al_PLOS COMP BIOL_Reply to reviewers.docx [file pcbi.1007818.s031.docx]

**Reply to reviewers:**

*Reviewer #1: The manuscript "Insights into adenosine A2A receptor activation through cooperative modulation of agonist and allosteric lipid interactions" by Bruzzese et al. describes the lipid-dependent activation of the receptor. The authors have performed 2 microsecond atomistic MD simulations on 4 different systems: the apo and ligand bound inactive structure of the receptor in DOPC and DOPG bilayers, with 4 replicates for each system in consideration. Based on these simulations, the authors suggest that lipid effects and ligand binding affect receptor activation states. There have been several papers using simulations analyzing the A2A activation and the authors need to highlight salient features of their own work better. I have several comments that need to be taken into consideration to strengthen the manuscript.*

*1. Is the entry of a DOPG molecule into the lumen of the receptor an artifact of the system setup and the micro-system with large electrostatic effects (charged lipids etc.)? Bilayers of only PG lipids are quite difficult to handle experimentally due to the large electrostatic repulsion between the lipids. The authors need to validate that this is not an artifact of their system set up and simulations. For instance what are the counter ions used? I would recommend using a PC/PG mixed bilayer to substantiate their findings.*

Counter ions were naturally used to neutralize the system (otherwise it would generate error messages and probably be too unstable to execute), as previously stated in methods: “Each receptor-membrane system was solvated with TIP3P water molecules above and below the membrane, with a concentration of 0.3 M KCl for zero system net charge”. Therefore, in the case of DOPG, K+ ions were used in sufficient numbers and in a suitable ratio with respect to Cl- ions in order to offset the negative charge of the membrane. This is a standard technique, which is automatically calculated during system set-up in the CHARMM-GUI web-server, which we have implemented in our previously published studies e.g. “Bruzzese A, Gil C, Dalton JAR, Giraldo J. Structural insights into positive and negative allosteric regulation of a G protein-coupled receptor through protein-lipid interactions. Sci Rep 2018; 8: 4456”. In this particular study, we tested three types of lipid membrane in relation to the Beta-2 adrenergic receptor: DOPC, DOPE and DOPG. We found each of these membranes to meet expected physical characteristics and we were able to replicate experimental results in terms of protein-lipid allosterism (“Dawaliby, R., Trubbia, C., Delporte, C., Masureel, M., Van Antwerpen, P., Kobilka, B. K. & Govaerts, C. Allosteric regulation of G protein-coupled receptor activity by phospholipids. Nat Chem Biol, 2016, 12, 35-39”). We therefore have little reason to doubt the integrity of DOPC or DOPG membranes in our current study, especially as we follow the same protocol as before and because we see no anomalies or unusual behaviour in any of our MD systems. Regarding the entrance of a phospholipid into the G protein binding-site (intracellular lumen) of a GPCR, this was first reported by a different laboratory in the paper: “Neale C, Herce HD, Pomès R, García AE. Can Specific Protein-Lipid Interactions Stabilize an Active State of the Beta 2 Adrenergic Receptor? Biophys J. 2015; 109(8): 1652-1662”. In this study, the Beta-2 adrenergic receptor was simulated in an active state (minus its G protein) rather than the inactive. It was reported that both neutral and anionic phospholipids (POPC or POPG) were observed entering the G protein binding-site (intracellular lumen), although anionic lipids were much more likely to do so than neutral lipids. The reported reason for this is that PG lipids have a smaller hydrophilic head-group, which can more easily access the gap between TM6 and TM7 in an activated receptor conformation than the bulkier hydrophobic head-group of PC lipids. Interestingly, we also observed similar findings in our recent study of the CB1 receptor [Díaz Ó, Dalton JAR, Giraldo J. Revealing the Mechanism of Agonist-Mediated Cannabinoid Receptor 1 (CB1) Activation and Phospholipid-Mediated Allosteric Modulation. J Med Chem. 2019; 62(11): 5638-5654] where we used two types of membrane: DOPG and POPC. Even though we were able to fully activate CB1 in both membrane types, we observed that receptor activation was more consistent in DOPG membranes and that DOPG lipids were more likely to enter the G protein binding-site of the activated receptor than POPC lipids. In terms of our current study on the adenosine A2A receptor, there are several factors that determine the results that we see. Firstly, unlike with CB1, we are only able to fully activate the adenosine A2A receptor in a DOPG membrane (i.e. not in DOPC or POPC), which suggests protein-lipid allosterism is crucial for this receptor. Therefore, it makes sense that we are more likely to see lipid entrance in these DOPG simulations where the gap between TM6 and TM7 is at its largest. Secondly, as previously stated, DOPG has a smaller lipid head-group than DOPC, so it is more likely for it to enter the G protein binding-site when the receptor is in a compliant state. Thirdly, even though an intermediate receptor conformation is obtained in a DOPC membrane (and POPC; see below for details) with bound agonist (adenosine or NECA), entrance of DOPC (or POPC) into the G protein binding-site is much more difficult because of its bulkier size. It is therefore not observed in the replicas we perform here. Presumably, if full receptor activation were captured in a DOPC (or POPC) membrane, for example in much longer MD simulations, then phospholipid entry would likely be observed eventually. However, this is a hypothesis we are currently unable to test. We have however modified the methods section to more clearly describe the process of adding K+ counter ions to neutralize system charge in DOPG membrane systems on p44:

“Each receptor-membrane system was solvated with TIP3P water molecules above and below the membrane, and an overall concentration of 0.3 M K+Cl- with ratio of positive/negative ions adjusted accordingly (automatically during CHARMM-GUI system setup) in order to maintain net zero charge in each system.”

Finally, regarding the concept of implementing hybrid DOPG/DOPC membranes, we appreciate the suggestion by the reviewer and we understand their point of view, however our past experience informs us that this would likely create greater variation in the system and increased uncertainty in terms of biological effect. This would make analysis and interpretation more difficult, not less. Our preference is therefore to avoid this scenario and only analyse homogeneous membranes so that our results are as clear and consistent as possible. Instead, we have chosen to perform and discuss extra MD simulations under other conditions as suggested by the reviewer. This includes addition of an extra agonist (NECA) and extra control simulations in a third type of homogeneous membrane (POPC). In summary, we think heterogeneous membranes are better suited for future studies. We hope the reviewer agrees.

*2. One major weak point that I see is that the lipid is suggested to act as an allosteric modulator, without showing specific (converged) binding sites and discussing allosteric communication pathways. Otherwise these could be simple non-specific "environment effects". Calling it an allosteric effect implies a specific allosteric binding and long-range communication pathways from that site to either ligand binding or G-protein coupling site. In this regard, the allosteric behavior would need to be more rigorously shown.*

We agree with the reviewer that allosterism should be viewed in terms of specific interactions, rather than non-specific “environmental effects”. Several specific intracellular protein-DOPG interaction sites were identified in our original submission and displayed in Fig 6 and S11 Fig (NB: these two figures have now been merged to make a new version of Fig 6). As shown in Fig 6, in both apo and adenosine-bound states, four converged DOPG hotspot binding sites were identified, which were previously described in the manuscript in terms of: “areas between TM1 and H8, between TM3 and TM4, and on the respective surfaces of TM5 and TM6”. Several specific protein-lipid interactions in these hotspots were shown in S11 Fig and are now included in new Fig 6. In particular, this involves DOPG interaction with two tryptophan residues at the bottom of TM1, DOPG interaction between two adjacent arginine residues located on ICL2 and TM4, DOPG interaction with two arginine residues on TM5, and DOPG interaction between lysine and histidine residues on TM6. Furthermore, all these protein-lipid interactions can be observed in the high-resolution crystal of adenosine A2A in its inactive state where oleic acid takes the place of DOPG (PDB id: 4EIY), although some differences can be observed in the way oleic acid interacts with TM6 where the conformation of the receptor is perhaps not conducive (i.e. inactive as opposed to active). A more detailed description of these protein-lipid interactions has now been added to the manuscript on p21:

“In these sites, specific protein-lipid H-bonds and electrostatic interactions can be identified, including DOPG interaction with: i) a pair of tryptophan residues at the intracellular side of TM1 (W29 1.55 and W32 1.58), ii) neighbouring arginines on TM4 and ICL2 (R111 34.52, R120 4.41), iii) a pair of arginines on TM5 (R199 5.60, R206 5.67), iv) a lysine-histidine couple on TM6 (H230 6.32, K233 6.35). Each of these protein-lipid interactions is observed across all replicas in a DOPG membrane and marked by particularly stable lipid binding modes. For example, in several trajectories, a single DOPG molecule closely interacts with residue H230 6.32 continuously for 2 µs, or in others, one lipid is exchanged for another after 500-1000 ns and the interaction is re-established.”

Furthermore, in order to better convey these key intracellular protein-DOPG interactions, we have simplified the structural images previously shown in S11 Fig to only show protein-lipid interactions with especially high specificity (please see new Fig 6). We believe these interactions can be called “allosteric”, in particular the latter interaction on TM6 with H6.32 and K6.35, because they help to stabilize key TM helix conformations required for receptor activation, especially in terms of an outward position of TM6. For this reason, we have performed an extra analysis of DOPG lipid binding to H6.32 on TM6 (see extra SI figure: S13 Fig). In terms of TM6 conformational change, this is directly coupled to the G protein binding-site (now shown in Fig. 11) and in terms of longer-range effects, via core residues on TM6, such as selected positions of W6.48 (S8 Fig) affecting solvation in the receptor interior (Fig. 9), which in turn modifies the stability of bound agonist in a DOPG membrane (Fig. 8). We believe this fits a proper description of allosteric behaviour in two different directions (intracellular and extracellular), although we understand our initial characterization was perhaps not thorough enough due to its inherent complexities and concerns about word/figure limits. We have therefore modified the manuscript in the following sections to better describe these findings:

On p26-27: “In light of these effects on ligand stability and extracellular conformational changes, adenosine-bound A2aR displays the same four protein-DOPG allosteric interaction hot spots as seen with the apo state in DOPG: TM1-H8, TM3-4-ICL2, TM5, and TM6 (Fig 6 B,C). Closer inspection reveals the same specific intracellular protein-lipid interactions as observed in the apo state (Fig 6 D-G), but with increased stability of interaction between the lysine-histidine couple on the intracellular surface of TM6 (H230 6.32, K233 6.35) and a DOPG molecule (S13 Fig). This is suggestive of an allosteric pathway acting through TM6 for two-way communication between bound agonist in the orthosteric pocket and bound intracellular lipids.”

On p28: “However, only in replicas #2 and #4, does TM6 move outwards enough to establish a TM3-TM6 distance that matches the active crystal state (maximum values of 18.2 Å and 20.4 Å, respectively) within the final 400 ns of each replica (Fig 8 G). This degree of conformational change corresponds to a maximum TM6 RMSD of 7.8 Å and 8.4 Å, and a minimum of 2.3 Å compared to the active crystal (S6 Fig). This degree of movement can be directly correlated with particularly stable DOPG binding to the intracellular surface of TM6 via positively charged residues: H230 6.32 and K233 6.35 (S13 Fig).”

On p36-37: “As such, there is a correlation between sustained binding of PG lipids to intracellular regions of TM6 and enhanced TM6 conformational change, resulting in more efficient G protein docking, rearrangement of the internal water-mediated polar network, tryptophan sidechain toggling, more stable protein-agonist H-bonding, and increased stabilization of ECL2. This creates a multidirectional allosteric network where different entities affect the action of others.”

*3. Is DOPC a correct membrane control ? The high fluidity and curvature of the system is perhaps not a good control of a"generic membrane". In fact the authors at one point write that it can be compared to POPC, a true "representative" lipid. This statement should be modified and the authors to justify the use of DOPC/DOPG.*

Although POPC is traditionally used for control purposes, as in our previously published study of CB1 where effects of DOPG and POPC membranes were compared [Díaz Ó, Dalton JAR, Giraldo J. Revealing the Mechanism of Agonist-Mediated Cannabinoid Receptor 1 (CB1) Activation and Phospholipid-Mediated Allosteric Modulation. J Med Chem. 2019; 62(11): 5638-5654], we believe that for this particular study on adenosine A2A receptor, DOPC was a more suitable control lipid because it contains exactly the same fatty acid chains as DOPG, meaning that differences observed between membranes can be attributed to lipid head groups exclusively and their polar/electrostatic interactions with the protein, rather than unpredictable effects elicited by differentially saturated or unsaturated fatty acid chains. We observed no issues with the fluidity or curvature of the DOPC system in particular, although we appreciate the reviewer’s expert opinion. Indeed, with bound adenosine in a DOPC membrane, we observed reassuringly consistent protein behaviour in terms of reaching an intermediate receptor state (consistent with the adenosine-bound A2A crystal structure). This is very similar to the extra duplicated control simulations we have now performed in a POPC with bound adenosine (S12 Fig, S25 Fig, S26 Fig). Likewise, in the apo state in a POPC membrane, the receptor remains inactive, just like in DOPC. This supports the notion that MD simulations performed in DOPC and POPC membranes are indeed comparable. Furthermore, in our previously published study of the Beta-2 adrenergic receptor [Bruzzese A, Gil C, Dalton JAR, Giraldo J. Structural insights into positive and negative allosteric regulation of a G protein-coupled receptor through protein-lipid interactions. Sci Rep. 2018; 8(1): 4456], we used a DOPC membrane as a control (rather than POPC) because we wanted to closely replicate published results in experimental nanodiscs (“Dawaliby, R., Trubbia, C., Delporte, C., Masureel, M., Van Antwerpen, P., Kobilka, B. K. & Govaerts, C. Allosteric regulation of G protein-coupled receptor activity by phospholipids. Nat Chem Biol, 2016, 12, 35-39”), which we did, including consistent protein behaviour. We therefore have little reason to doubt the suitability of a DOPC membrane as a control, particularly as it possesses the same lipid head group as POPC, which is why we made the chemical comparison between DOPC and POPC in the manuscript. We believe this comparison to be reasonably fair, although as we have added a new POPC section on p18-19, we have chosen to remove the sentence entirely and replace it with:

“As an extra control, additional MD simulations were performed in duplicate in a POPC membrane with or without bound adenosine (summarized in S12 Fig). Like in DOPC, adenosine displays a similar level of positional instability when bound to the receptor in POPC (S12 Fig). Nevertheless, in both replicas the receptor is observed to reach an intermediate conformation similar to that observed in DOPC with bound adenosine (S12 Fig). This can be contrasted with the apo state in POPC where the receptor is observed to remain in an inactive conformation in both replicas and ECL2 is observed to move towards the empty orthosteric pocket by up to 2 Å (S12 Fig) in a similar manner to that observed with the apo state in DOPC. This suggests that DOPC and POPC membranes have similar effect (or lack of it) on A2aR with or without bound agonist, as might be expected.”

We have also added more detail on DOPC and DOPG fatty-acid chains and why DOPC is a suitable control for this study on p9:

**“**To this aim, we have chosen to study the agonist-mediated activation process of A2aR by performing quadruplicated unbiased microsecond-length MD simulations with bound agonists adenosine or 5'-N-ethylcarboxamidoadenosine (NECA) in two different homogeneous lipid environments: DOPC or DOPG (Fig 1), respectively. These two phospholipids contain the same 18:1 fatty-acid chains so are ideally suited for determining the allosteric effect of different lipid head groups on the receptor.”

*4. I would suggest that in addition to presenting the results as time evolution (of the distances), they are represented as population distributions to have a better understanding of the contribution of different conformational states.*

We thank the reviewer for the suggestion. We have added new population distribution plots to S24 and S25 Figs, which are independently measured according to TM3-TM6 (ionic-lock) and TM3-TM7 distances. Furthermore, we have updated old Fig 11 (which has become Fig 10 in the new version) to now incorporate a 3D plot of these two key distances plotted against each other and receptor conformation frequency represented as heat intensity. This gives a strong indicator of the total distribution of different receptor conformations (i.e. inactive, intermediate, actives) obtained in our MD simulations. We hope the reviewer approves. Discussion of these new figures can be found on p32:

“The cumulative effects of bound phospholipids and agonist are observed to have profound consequences on the overall population of receptor conformations, which can be defined according to key inter-helical TM3-TM6 and TM3-TM7 distances (Fig 10, S24 Fig, S25 Fig). Furthermore, MD simulations with the more potent NECA agonist clearly reveal that agonist-mediated receptor activation results in approximately equal formation of two distinct active-like receptor conformations, as previously proposed by NMR experiments [Prosser *et* *al*. Bioessays. 2017; 39(9)]”.

*5. The authors have extensively used RMSDs as their major indicator to identify conformational states at the start of the paper. I suggest authors use other collective variables (such as what they have calculated later, ionic lock, TM6-TM2 distance etc.) to make this more robust.*

In the main manuscript, we have included RMSD plots for the ligand in order to measure the position of the agonist, which is an important feature, whilst RMSD plots of the protein and RMSDs of individual helices are mostly reserved for SI, although we do mention specific RMSD values in the main text where we think it is appropriate. This is because RMSD is a universally recognized measurement for protein conformational change across a wide variety of protein studies and excluding it might result in criticism from general readers. However, we certainly recognize the inherent limitations of RMSD for measuring GPCR conformations, which is why we focus mainly on TM3 and ECL2 vertical displacement distance (not RMSD) and inter-helical internal distances (such as ionic-lock, TM3-TM7 and TM3-TM5) in each results section throughout the manuscript and in our main figures. We think these are indeed better suited for understanding the activation state of the receptor rather than RMSD. As a result, we have added population distribution plots as suggested in point (4) above, which reveal the frequencies of different conformations of the receptor according to specific internal distances (not RMSD). Finally, we have replaced old Fig 11, which was previously a blend of RMSD and ionic lock distances, to now be based solely on internal distances: ionic lock and tm3-tm7 distances. We think this is a better figure and is no longer subject to RMSD-based criticisms (please see new Fig 10).

*6. Statistical and sampling errors need to be presented for all plots.*

We thank the reviewer for this point. As a consequence, we have included new boxplots corresponding to key measurements used for assessing protein conformational change in our main figures. These include ionic-lock and TM3-TM7 distances, as well as ECL2 and TM3 vertical movements. Please see new S26 Fig for details.

*7. There are a large number of papers that have identified cholesterol binding sites on this receptor. Since cholesterol is an important constituent of the membrane where the receptor resides, the authors should discuss their results in the context of cholesterol binding. If specific cholesterol binding sites are that critical for the receptor, wouldn't these results be misleading? Perhaps a mixed bilayer (with cholesterol) might be more relevant. In the absence of such simulations, the authors need to discuss these points better. There have been reports of cholesterol in the receptor lumen for this receptor. Does it replace DOPG in the lumen or is it cooperative binding?*

We presume the reviewer is referring to “Guixà-González R, Albasanz JL, Rodriguez-Espigares I, Pastor M, Sanz F, Martí-Solano M, Manna M, Martinez-Seara H, Hildebrand PW, Martín M, Selent J. Membrane cholesterol access into a G-protein-coupled receptor. Nat Commun. 2017; 8: 14505”, where cholesterol is observed to transiently enter the orthosteric pocket of apo A2aR at relatively low frequency (observed in 3/40 replicas), through an extracellular gap between TM5 and TM6, when the receptor is embedded in a cholesterol-rich heterogeneous membrane and without bound agonist. This is quite different from our observations of physically stable protein-phospholipid allosteric interactions at the intracellular side of the receptor in a homogeneous membrane with bound agonist, and which are observed across all our replicas, either on the receptor surface or inside the G protein binding site (intracellular lumen). We do not observe any phospholipids entering the orthosteric pocket or making specific physically-stable extracellular interactions. We are aware of reports of phospholipids making interactions between TM1 and TM7 on the extracellular side of certain GPCRs [Sengupta D, Prasanna X, Mohole M, Chattopadhyay A. Exploring GPCR-Lipid Interactions by Molecular Dynamics Simulations: Excitements, Challenges, and the Way Forward. *J Phys Chem B*. 2018; 122: 5727–5737], although our observations suggest this extracellular protein-lipid interaction in adenosine A2A is not as stable as intracellular interactions or specific to either DOPG or DOPC/POPC lipids. On the other hand, there have been several reports of cholesterol binding adenosine A2A at several locations on the receptor surface, which can be viewed as analogous to the type of phospholipid interactions we observe in our study. These include predicted cholesterol binding sites at various extracellular or intracellular receptor locations. However, not all these predictions have been experimentally validated or their precise functional effects elucidated, which makes their discussion difficult. Concerning the most validated cholesterol binding spots: (i) the CCM (cholesterol consensus motif) region, located in a mostly hydrophobic cavity formed between TM1-TM2-TM4 at the intracellular side of the receptor is conserved across many class A GPCRs [Sarkar P, Chattopadhyay A. Cholesterol interaction motifs in G protein-coupled receptors: Slippery hot spots? Wiley Interdiscip Rev Syst Biol Med. 2020: e1481]. This is the same cavity where DOPC lipids are observed to cluster in our control MD simulations, which we previously refer to in our manuscript and show in Figure 6. It is therefore possible that cholesterol could compete with the binding of DOPC lipids at this location and favour receptor activation over inactivation, which is not necessarily desirable as a control for our study. However, it is relatively unlikely that cholesterol would compete with DOPG lipids here because the latter binds to an adjacent and distinct site formed between TM1 and H8, not with TM2. (ii) A novel cholesterol binding site has been proposed to exist on the extracellular surface of TM6 [Rouviere E, Arnarez C, Yang L, Lyman E. Identification of Two New Cholesterol Interaction Sites on the A2A Adenosine Receptor. Biophys J. 2017; 113(11): 2415-2424] and this has been validated by high-resolution inactive crystal structure of adenosine A2A (PDB id: 4EIY). However, its precise function is unclear. It is also unlikely to affect intracellular binding of DOPG or DOPC lipids. iii) From the same paper, a second cholesterol binding site has been predicted to exist in a crevice between TM5 and TM6 at the intracellular side of the receptor. This was based on MD simulations of the receptor in an antagonist-bound inactive state so it is unclear whether this site is relevant to the active state of the receptor, or how cholesterol binding at this site might relate to receptor activation. To our knowledge, it has not yet been validated by receptor crystallization. However, it has been corroborated to some degree by a separate paper where a near-active conformation of adenosine A2A was obtained during an 80-microsecond MD simulation of the apo state, without bound agonist or simulation replicas, as far as we can tell [Lovera S, Cuzzolin A, Kelm S, De Fabritiis G, Sands ZA. Reconstruction of apo A2A receptor activation pathways reveal ligand-competent intermediates and state-dependent cholesterol hotspots. Sci Rep. 2019; 9(1): 14199]. In theory, cholesterol binding at this site could potentially enhance or compete with intracellular DOPG binding on the surfaces of TM5 and/or TM6, which our study shows to be important for full receptor activation. Therefore cholesterol may not be ideally suited for studying such processes. Of all the predicted cholesterol binding sites, it is the CCM region between TM1-TM2-TM4 where cholesterol is most reliably predicted to favourably bind the active receptor state over the inactive, which appears to be the primary route whereby cholesterol might positively modulate adenosine A2A [McGraw C, Yang L, Levental I, Lyman E, Robinson AS. Membrane cholesterol depletion reduces downstream signaling activity of the adenosine A(2A) receptor. Biochim Biophys Acta Biomembr. 2019; 1861(4): 760-767]. It would therefore imply that cholesterol competition with DOPC at this site is more important than potential interactions with DOPG. Finally, the electrostatic interaction that mediates DOPG binding at the intracellular G protein cavity is formed via residue Arg3.50 (part of the ionic lock), which cholesterol would be highly unlikely to replicate because it does not contain a charged lipid head-group or inherent flexibility of phospholipid fatty-acid chains, which our results suggest are critical features. Furthermore, from our knowledge, nobody has suggested that cholesterol could make this type of intracellular interaction in GPCRs. Therefore, regarding these overall observations, we think it is unnecessary to include cholesterol in our study, particularly as it has been investigated before and because it has not been proven to be strictly necessary for adenosine A2A activation (merely that its absence may reduce receptor activity in some instances, as shown by McGraw et al, 2019). As one of our main objectives is to determine allosteric effects of phospholipids on adenosine A2A, which we feel is an under-reported but important phenomenon in GPCRs, mixing cholesterol in this study could confuse our findings. This is further complicated by the matter that cholesterol binding to GPCRs is a relatively slow, weak process [Cang X, Du Y, Mao Y, Wang Y, Yang H, Jiang H. Mapping the functional binding sites of cholesterol in β2-adrenergic receptor by long-time molecular dynamics simulations. J Phys Chem B. 2013; 117(4): 1085-94], which probably requires even longer simulations than we have performed, thus making statistical comparisons difficult. This is demonstrated by the longer MD simulations previously used to predict cholesterol binding in adenosine A2A, i.e. six microseconds but with no replicas in “Rouviere E, Arnarez C, Yang L, Lyman E. Identification of Two New Cholesterol Interaction Sites on the A2A Adenosine Receptor. Biophys J. 2017; 113(11): 2415-2424”. In totality, we hope the reviewer sees our point of view that cholesterol is not required to properly investigate agonist-mediated activation of adenosine A2A or allosteric modulation by phospholipids. We have however updated the manuscript to include some of these points in a slightly wider cholesterol-based discussion. For example on p22 and p36:

“Interestingly, the TM1-TM2-TM4 intracellular cavity is recognized as containing the cholesterol consensus motif or CCM, which in active A2aR appears to favourably bind membrane cholesterol and could account for its experimentally observed enhancement of receptor signalling. Interestingly, in our MD simulations, this cavity instead sequesters DOPC phospholipids, which might provide an opposite effect to cholesterol, potentially suggesting competition between these two molecules. In terms of comparison between DOPG and cholesterol, the binding of phospholipids to the intracellular surfaces of TM5 and TM6 may potentially share similarities with a reported second intracellular binding-site of cholesterol in a hydrophobic crevice between TM5 and TM6. As cholesterol is an uncharged molecule, its precise mode of interaction is likely different to the electrostatic nature of anionic DOPG, meaning these two molecular species might theoretically be able to co-bind the receptor in this area. However, the exact function of cholesterol binding in this location in terms of receptor activation is difficult to verify as it was observed within MD simulations of the antagonist-bound inactive receptor (which remains inactive) and because crystal structures of active A2aR lack co-crystallized cholesterol.”

“Through its binding, cholesterol has been reported to stabilize the active receptor conformation. This poses the possibility of PC lipids and cholesterol competing at the same location and exerting different influences over receptor activity. Likewise, cholesterol has been suggested to also bind between TM5 and TM6, which could conceivably potentiate allosteric effects of PG lipids in this area. Although an intriguing question, cholesterol binding is beyond the scope of this study, which as a relatively weak and slow process (compared to phospholipids) would necessitate much longer MD simulations and preclude execution of quadruplicates. In terms of protein-phospholipid interactions on the extracellular side of the receptor, we also observe some binding of phospholipids in the hydrophobic interface between TM1 and TM7 as has been previously proposed in some GPCRs [Sengupta *et* *al.*, J Phys Chem B. 2018; 122(22): 5727-37], however in our MD simulations these are not as physically stable as interactions at the intracellular side (hence relative lack of clustering intensity in Fig 6) and are seemingly not specific to either PC or PG lipids.”

*8. The authors need to provide more details on the MD parameters used: pressure coupling, temperature coupling, handling of electrostatics etc. for completeness. If there are word restrictions, perhaps the details can be provided in the Supplementary.*

We have updated methods to include these details on p44:

”Briefly, each receptor-membrane system was equilibrated for 28 ns at 300 K (Langevin thermostat) and 1 atmosphere (Berendsen barostat) using a 4 fs time step and electrostatics cut-off of 9.0 Å.”.

*9. A homogeneous negatively charged bilayer would introduce electrostatic artifacts in the system and could largely affect the protein behavior. How is this taken care of - since the system does not appear to have neutralizing counter-ions added. Similarly, where are these lipids actually located in the bilayer- which leaflet of the bilayer. In this regard, are the results consistent with the known lipid abundance?*

As previously mentioned in point (1), K+ counter ions were used to neutralize the system, which we have successfully used before in previous publications to offset the negative charge of the DOPG membrane. We have modified the methods section to more clearly describe this process on p43:

“Each receptor-membrane system was solvated with TIP3P water molecules above and below the membrane, and an overall concentration of 0.3 M K+Cl- with ratio of positive/negative ions adjusted accordingly (automatically during CHARMM-GUI system setup) in order to maintain net zero charge in each system.”

Finally, both leaflets of the DOPG membrane are the same i.e. homogeneous. We believe this is the fairest approach for obtaining consistency and avoiding competing or unpredictable biological effects that might arise from heterogeneous membranes. In terms of abundance, as previously published, dioleoyl (DO) fatty acids are highly present in human membranes, accounting for ~30% of all fatty acids in the brain [Abbott SK, Jenner AM, Spiro AS, Batterham M, Halliday GM, Garner B. Fatty acid composition of the anterior cingulate cortex indicates a high susceptibility to lipid peroxidation in Parkinson's disease. J Parkinsons Dis. 2015; 5(1): 175-85], and are certainly of biological relevance [Bruzzese A, Gil C, Dalton JAR, Giraldo J. Structural insights into positive and negative allosteric regulation of a G protein-coupled receptor through protein-lipid interactions. Sci Rep. 2018; 8(1): 4456]. On the other hand, although DOPG lipids specifically have relatively low biological expression (<2%), they are typically enriched around GPCRs like beta-2 adrenergic with values such as >7% or >16% if including all PG lipids (Dawaliby, R., Trubbia, C., Delporte, C., Masureel, M., Van Antwerpen, P., Kobilka, B. K. & Govaerts, C. Allosteric regulation of G protein-coupled receptor activity by phospholipids. Nat Chem Biol, 2016, 12, 35-39). This indicates their potential importance in GPCR function, which we have been able to independently verify with studies on beta-2 adrenergic and CB1 receptors [Díaz Ó, Dalton JAR, Giraldo J. Revealing the Mechanism of Agonist-Mediated Cannabinoid Receptor 1 (CB1) Activation and Phospholipid-Mediated Allosteric Modulation. J Med Chem. 2019; 62(11): 5638-5654], as well as adenosine A2A in this current study.

*10. Line 220 : “Moreover, the distance between TM3 and TM7 is decreased, most noticeably between intracellular residues R102 3.50 and Y288 7.53, while the distance between TM3 and TM5 is increased, exemplified by residues R102 3.50 and Q207 5.68 (S1C-E Fig) (S2D-F Fig) (S1 Table).” The sentence could be better discussed with the actual values of these distances to understand the magnitude of change in the distances. Typo in the table reference. It should be SI Table 2. Please check the consistency of the BW numbering as a superscript throughout the manuscript and for figure legends too.*

We have added distances to this sentence. On p11:

“Moreover, the distance between TM3 and TM7 is decreased, most noticeably between intracellular residues R102 3.50 and Y288 7.53, which are 5.0 Å closer in the active state, while the distance between TM3 and TM5 is increased, exemplified by residues R102 3.50 and Q207 5.68, which are 2.7 Å further away in the intermediate state (S2, S3 Fig) (S2 Table).”

*11. Line 223: “The main difference between intermediate and active crystal structures can be observed in the distance between TM3 and TM6 and intracellular ionic-lock residues R102 3.50 and E228 6.30, respectively. All these observations seem qualitative, referring to the actual value of the distance while describing in the results would be helpful.*

We already give the corresponding distances in the subsequent sentence on p12: “However, TM3-TM6 distance reaches 18.6 Å in the active, but only 9.9 Å in the intermediate crystal structure (S2 Fig, S3 Fig) (S2 Table).”

*12. Line 225: “Both crystals have TM6 oriented outwards compared with the inactive crystal structure. However, TM3- TM6 distance reaches 18.5 Å in the active, but only 9.9 Å in the intermediate crystal 228 structure (S1E and S2F Fig) (S2 Table)”. >> It’s 18.3Å, as mentioned in the table by authors - I guess 0.2Å makes a difference? Or put it as approx. >> Please refer to the table in the SI consistently: Table SI 2.*

We thank the reviewer for spotting this inconsistency. The table previously contained an error. We have corrected the sentence and table to 18.6 Å. Regarding format, PLOS requires supplementary tables and figures to be referred to as Sx Fig or Sx Table, where x=1-99.

*13. Line 244: “Over the course of these replicas, ECL2 continues to fluctuate up to a maximum of 8.6 Å and 10.9 Å RMSD, respectively (S3A Fig).” >> It appears that except for DOPC-apo simulations (replicate #2 and #3 ), all others - even for DOPG are comparable, indicating there is no difference in simulations with different bilayers. Why do you think that is? Is it possible it is not well equilibrated? Or converged? Statistical significance should be provided.*

The respective MD simulations mentioned by the reviewer are suitably well equilibrated (28 ns in each case is ample in our experience). ECL2 is a very long extracellular loop of 30 residues, which is partially stabilized by internal disulphide bonds. Nevertheless, in adenosine A2a as in Class A GPCRs generally, it is a highly flexible region, which interacts with external solvent and in normal situations is prone to conformational fluctuations, which allow ligand entry and exit or formation of transient interactions with other extracellular receptor features, including formation of a lid over the orthosteric pocket [Woolley MJ, Conner AC. Understanding the common themes and diverse roles of the second extracellular loop of the GPCR superfamily. Mol Cell Endocrinol. 2017; 449: 3-11]. As ECL2 is known to play a direct role in ligand binding and specificity in adenosine A2a in particular [Seibt BF, Schiedel AC, Thimm D, Hinz S, Sherbiny FF, Müller CE. The second extracellular loop of GPCRs determines subtype-selectivity and controls efficacy as evidenced by loop exchange study at A2 adenosine receptors. Biochem Pharmacol. 2013; 85(9): 1317-29], it is perhaps no surprise that when adenosine is bound in a DOPC membrane here, loop fluctuation is noticeably less compared to the apo state (e.g. see Fig S4). However, when no ligand is bound and the receptor remains in an inactive state as occurs in a DOPC membrane (or POPC as observed in our extra MD simulations, see S12 Fig), the orthosteric pocket remains wide (as indicated by larger TM3-TM7 distances), which potentially allows ECL2 to move partially downwards towards the orthosteric pocket by 2-4 Angstroms, forming a lid over the binding-site (but not always because of dynamic protein-water interactions). This is what accounts for the relatively high RMSDs for ECL2 recorded in replicas #2 and #3 in the apo state in a DOPC membrane. This alternative loop position does not appear to be so stable because only replica #2 maintains this downwards-like position, while in replica #3, ECL2 gradually moves back up towards its original ligand-bound crystal state. Likewise, in our extra POPC simulations, we see a similar process (S12 Fig). We understand that our original statement does not make this so clear. This section on p12-13 has now been expanded to:

“In replicas #2 and #3, a conformational change occurs (at 400 ns and 1000 ns, respectively) where ECL2 moves downwards by 2-4 Å to form a lid over the orthosteric pocket (Fig 3 A-F). Over the course of this process, ECL2 reaches a maximum of 8.6 Å and 10.9 Å RMSD, respectively (S4 Fig). However, in replica #3, the loop moves back towards its original position, away from the orthosteric pocket. This shows alternative conformations of ECL2 are possible but not necessarily physically stable. Nevertheless, in replica #2, the loop remains in a downwards position partially covering the top of the orthosteric pocket (Fig 3 A), albeit with conformational fluctuations (S4 Fig). This conformational change of ECL2 is possible in the apo state due to the lack of bound agonist and because the receptor remains inactive, thus generally maintaining the TM3-TM7 starting distance of 16.3 Å (Fig 3 D). This allows the latter section of ECL2 to adopt a lower position between the extracellular ends of TM3 and TM7. However, in the other two replicas, ECL2 broadly maintains its original crystal structure position (average RMSD across replicas #1 and #4 of 2.4 Å ±0.7 S.D.) (S4 Fig), which demonstrates ECL2 variability as previously discussed in class A GPCRs, especially in the apo state”.

The remarkable feature in this study is not so much that ECL2 experiences varying degrees of conformational fluctuation in the apo state in a DOPC membrane but rather that it undergoes much stricter behaviour in a DOPG membrane even when no ligand is bound (S4 Fig). This is because intracellular protein-lipid allosteric effects observed in a DOPG membrane (via hot spots at intracellular receptor regions: TM1/H8, TM3/ICL2/TM4, TM5 and TM6) facilitate conformational changes in TM helices which affect the orthosteric pocket and behaviour of ECL2. For example, in the apo-state in a DOPG membrane, TM7 moves inwards towards TM3 (decreasing TM3-TM7 distance, Fig. 5), which narrows the orthosteric binding pocket (see peer-review Fig 1 below). This is a phenomenon which has been observed before with adenosine A2A crystal structures with bound agonists (Dalton JA, Lans I, Giraldo J. Quantifying conformational changes in GPCRs: glimpse of a common functional mechanism. BMC Bioinformatics; 16: 124). However, it is observed here in the apo state because TM6 is encouraged to move outwards as the receptor activates, making space in the helical bundle for inward movement of TM7. This narrowing of the orthosteric pocket between TM3 and TM7 prevents ECL2 from moving downwards. As a result, ECL2 remains near its original crystallized position. In addition to TM7 conformational change, intracellular phospholipid allosteric binding between TM3, TM4 and ICL2 (shown previously in S11 Fig but now in new Fig 6) helps to stabilize TM4 conformation, which is normally a relatively loose helix on the edge of the helical bundle, being flanked by two long flexible loop regions: ECL2 and ICL2. This extra stabilization of TM4 according to RMSD (see peer-review Fig 2 below) helps to stabilize ECL2 in its original crystal conformation in a DOPG membrane. In other words, there is evidence of multiple allosteric pathways that connect intracellular and extracellular regions of the adenosine A2A receptor. In terms of significance of ECL2 movements, we also attach a boxplot for the reviewer’s attention (see peer-review Fig 3 below), as well as including it in new S26 Fig. This plot reveals a higher position of ECL2 when a ligand is bound (or lower position in apo), as well as enhanced conformational restriction in a DOPG membrane.

Peer-review Fig 1: Orthosteric pocket measurement in MD simulations of apo adenosine A2a (ECL2 not displayed). (A) In DOPC, replica #2, 2000 ns; B) in DOPG, replica #1, 2000 ns, C) in DOPG, replica #4, 400 ns.

Peer-review Fig 2: RMSD of TM4 in apo state in DOPC (left) and DOPG (right) membranes.

Peer-review Fig 3: Boxplot of vertical movement of ECL2 in different membrane environments (DOPC/POPC/DOPG), with/without bound adenosine (adn). MD simulations performed in quadruplicate for DOPG/DOPC and duplicate for POPC.

*14. Line 247: “However, in other replicas, ECL2 shows less conformational change (average RMSD across replicas #1 and #4 of 2.4 Å ±0.7 S.D.)” (Figure S3A). >> In figure S3B, there appears to be no line corresponding to the active crystal structure. Could the authors clarify?*

Both lines corresponding to inactive and active crystals are actually displayed in Fig S3B (now S4 Fig). However, because both these crystal structures contain ligands, their respective ECL2 positions are similar. This means that their Z-axial positions are also similar, hence the two lines overlap. In terms of our current study, clear differences in ECL2 behaviour are observed for apo versus ligand-bound receptor states but because there are no crystal structures of apo adenosine A2A receptor available, these findings are difficult to validate. As stated previously in point (13), because of protein-DOPG allosteric interactions (TM3/4/ICL2, TM5, TM6), behaviour of ECL2 is different compared to DOPC, especially when the receptor is in its apo state.

*15. Line 248 : Despite these conformational fluctuations of ECL2, little conformational change is observed in the receptor as a whole across all four replicas with an average RMSD of 2.5 Å ±0.6 S.D. (S4A Fig) or in key helices such as TM3 (average Z-axis displacement of 0.2 Å ±0.6 S.D., Fig 3G). >> Here I feel, inclusion of Helix 8 is not necessary since TM helices are the main hub of structural changes. Also, helix 8 is a membrane associated helix and not TM helix. >> In case of Figure S4A and S4B, RMSDs in A) panel are higher than that of the panel B). Does this indicate the structures are more similar to active crystal structure than the inactive in both the bilayers? This needs to be discussed.*

The reviewer is correct that Helix 8 is not a transmembrane helix and perhaps not strictly required in RMSD calculations. We have therefore recalculated all TMD RMSDs to only include TM1-TM7. The results are however very similar to before, which suggests any conformational change in H8 was relatively negligible. Regarding S4 Fig (which is now S5 Fig), which has been readjusted accordingly, we would like to point out to the reviewer that different Y-axis scales were previously used in (A) and (B). As a result, it may have looked like RMSD was lower/higher between different panels when it was not really so. We have now updated this figure so that the scales in each panel start at 0, making their comparison easier. In terms of the apo state in DOPC, the receptor is closer (lower RMSD) to the inactive state than active (higher RMSD), as expected, while with bound adenosine in DOPC, the receptor is in an intermediate state, which is slightly shifted towards the active but still closer to the inactive in general terms. On the other hand, in the apo state in DOPG, the receptor is somewhere between inactive and active with relatively similar RMSDs, which suggests an enhanced intermediate state, while with bound adenosine in DOPG the receptor is closer to active (lower RMSD) than inactive (higher RMSD). These results are broadly as expected while taking into account the relative limitations of RMSD measurements.

*16. Line 265: “Despite some of these observed differences, TM7 regularly revisits its inactive crystal conformation across all four replicas, suggesting this state is at least meta-stable and in line with the receptor remaining inactive (Fig 3A, D)”. >> A population analysis of this distance variation might represent the results better and actually show the increased presence of this conformation in the total ensemble sampled. >> Also refer to figure 11.*

As already mentioned in point (4), we have included new receptor conformation population plots, including TM3-TM7 distance, which accurately reveal the frequencies of different receptor conformations observed in relevant MD simulation systems (S24 and S25 Figs). We thank the reviewer for this suggestion because it clearly shows differences between predominant receptor conformations in different MD simulations (membrane type, apo vs ligand etc). In addition, we have updated Fig 11 (which is now new Fig 10) to a heatmap comparison of ionic-lock vs TM3-TM7 distances, including labelling of inactive, intermediate and active crystal reference points. We think this is a very useful figure of different receptor populations identified in our MD simulations. We hope the reviewer agrees.

*17. Line 286: “In replicas #3 and #4, adenosine achieves a stable binding mode in the orthosteric pocket between helices TM3, TM6 and TM7, which is consistent with its crystallized pose in the A2aR intermediate crystal structure [93].” >> Please refer to S10 here.*

We assume the reviewer is referring to the alternative but unstable binding pose that adenosine finds in replicas #1 and/or #2, which is not consistent with its crystallized pose, as shown in S10 Fig. We already describe this process in subsequent sentences on p15:

“However, in replicas #1 and #2, adenosine changes its binding pose frequently without finding stability, in some cases moving up to a maximum of 8.9 Å. This is because adenosine is relatively small and is able to occupy an alternative space in the orthosteric pocket in between helices TM1, TM2 and TM7 (S10 Fig).”

We think this description and reference to S10 Fig is already adequate. We hope the reviewer agrees.

*18. Line 289: “At the same time, N253 6.55 forms a stable protein-ligand H-bond with average occupancy of 95.0% (±5.4 S.D.)”. >> Please clarify the residue number N6.55 or 6.33 and check S13 legend and figure.*

We thank the reviewer for finding this error. It should be N6.55. It has been corrected in S13 Fig (now S15 Fig).

*19. Please label the helices in Figure 6. Are the thickness plots averages over one set or 4 replicates?*

We have labelled helices in Fig 6, as requested. The lipid density plots are calculated over one replica of each membrane condition. Other replicas generally yield similar plots so we think this approach is fine. For example, we have attached an image below of replicas #1 and #2 for the apo receptor in a DOPG membrane. Despite differences in receptor conformation, DOPG hotspots are fairly similar in intensity and position.

Peer-review Fig 4: Comparison of average membrane thickness in two replicas of MD simulation of apo adenosine A2A receptor in DOPG membrane. (A) replica #1, (B) replica #2.

*20. Line 323: “Regarding key protein-ligand interactions, N253 6.33 located on TM6 and S277 7.42 and H278 7.43 located on TM7 make H-bonds with adenosine with average occupancies of 92.1% (±7.0 S.D.), 49.5% (±25.2 S.D.) and 62.9% (±16.1 S.D.) (S9B Fig)”. >> It should be Figure S9C not S9B. >> also please provide error bars on the plot.*

We thank the reviewer for finding these inconsistencies. “N253 6.33” should be “N253 6.55”. It should also indeed be Figure S9C, not Figure S9B. We have corrected these errors and updated S9 Fig (now S11 Fig). In terms of providing error bars, we have added extra plots to both S9 and S13 Figs (now S11 and S15 Figs), which now show the mean average H-bond occupancies per residue across all relevant replicas with corresponding S.D. error bars in each case. This is in addition to the previously included H-bond occupancies per residue per individual replica, which can be used to cross-check individual replicas against other figures where the receptor undergoes activation or not, so as to better reveal the most functional protein-ligand H-bonds mediating receptor activation.

*21. Line 332: “This precedes the gradual separation of the intracellular end of TM6 away from TM3 by a maximum of 5.1 Å, which starts at a distance of 8.3 Å and increases up to 13.4 Å within 0.7 μs of the same three replicas (Fig 4 G).” >> How do we know this isn’t a one off event ? Could the authors comment on the statistical significance?*

As this process of TM6 movement occurs in 3 out of 4 replicas within 700 ns in a DOPC membrane with bound adenosine, we are taking this as evidence that it is not a one off event. This is the fundamental reason for making four replicas: so that we can have confidence in our observations. Furthermore, we do not see this steady process occur in replicas of the apo receptor in DOPC where only 1 replica shows an equivalent increase in TM3-TM6 distance, but it is transitory and quickly fades. To assess the statistical significance of this dynamic event, firstly, output data were saved every 100 ns and each replica considered as an experimental unit. Then, we performed a two-way (ligand: adenosine, none; time) analysis of variance with repeated measures on the time factor (we have 7 measurements on the same replica). A p-value of 0.0492 was obtained for the ligand factor when the four replicas of adenosine-bound receptor were included in the analysis, which decreased to 0.0266 when only replicas #1, #3 and 4 were included. These results statistically confirm (at the p<0.05 level of statistical significance) TM6 outward movement with bound adenosine relative to apo. In addition, we have generated boxplots of appropriate inter-helical distances, including TM3-TM6 (please see attached plot below, as well as S26 Fig) across different simulation conditions, which confirms appreciable TM6 outward movement with bound adenosine in either DOPC or POPC membranes compared to the apo state in DOPC or POPC. We have also reworded the quoted section in the manuscript on p17 to:

“This precedes the gradual separation of the intracellular end of TM6 away from TM3 by a maximum of 5.1 Å, which starts at a distance of 8.2 Å and increases up to 13.4 Å within 0.7 μs across these same three replicas (Fig 4G). However, as this conformational change of TM6 occurs mainly at its intracellular end, RMSD of TM6 shows only moderate change, reaching 4.0 Å in replica #4 (S6 Fig).”

Peer-review Fig 5: Boxplot of TM3-TM6 (ionic lock) distance in different membrane environments (DOPC/POPC/DOPG) with/without bound adenosine (adn). MD simulations performed in quadruplicate for DOPG/DOPC or duplicate for POPC.

*22. “Our MD simulations suggest that ECL2 is likely to be more stabilized in a higher position in a DOPG environment rather than in DOPC, which appears to assist conformational change in TM3 as well as enhancing the stability of bound adenosine. On a speculative level, this influence of lipids on ECL2 may provide a structural explanation for the observation that GPCRs exhibit different ligand efficacies in different cell lines [108, 110]”. >> Why is ECL2 stabilized in a higher position? Is there a DOPG lipid binding at that site? Or are there long-range effects.This appears to be a central part of the paper and needs to be discussed better.*

Specific DOPG lipid binding does not occur at the extracellular side of the protein or in the orthosteric site. The only observed protein-lipid extracellular interaction of note occurs in the interface between TM1 and TM7 and is equally present for both DOPC/POPC and DOPG lipids. We therefore deem it to be a non-specific protein-lipid interaction. However, specific DOPG binding at several intracellular allosteric sites causes conformational changes in the protein that tends to favour an intermediate or active-like state of the receptor, which has a knock-on effect to the behaviour of ECL2. In an intermediate or active-like state, TM3 and TM7 are closer together, which narrows the orthosteric pocket as we observed in a previous study (Dalton JA, Lans I, Giraldo J. Quantifying conformational changes in GPCRs: glimpse of a common functional mechanism. BMC Bioinformatics; 16: 124). In turn, this prevents downward movement of ECL2 and stabilizes the loop near its original ligand-bound crystal structure position (i.e. upward). This mechanism is modulated by allosteric DOPG interactions with TM5 and TM6, which are encouraged to move outwards as the receptor activates, making space for inward movement of TM7. Likewise, the protrusion of a DOPG lipid into the G protein binding-site (intracellular lumen) between TM6 and TM7 favours an active-like receptor state that also encourages closer interaction between TM3 and TM7, narrowing the orthosteric pocket at the extracellular side. In addition to conformational changes of TMs 5-7, DOPG binding in an intracellular cavity between TM3, TM4 and ICL2 (shown in new Fig 6) helps to stabilize TM4 conformation (see point 13 above), which further stabilizes ECL2. Therefore the dynamic behaviour of ECL2 is affected by several long-range protein-lipid allosteric interactions, as well as by an agonist when bound. We have added extra content at several sections in the manuscript regarding the relationship between DOPG lipids and ECL2, including:

On p19: “This suggests that the extracellular side of the receptor behaves differently in an anionic membrane environment, which is related to conformational changes in the TM domain that contribute to conformational restriction of ECL2. In particular, this involves inward movement of TM7 (see below) that narrows the orthosteric pocket, which has previously been described in agonist-bound A2aR crystal structures.”

On p20: “These initial intracellular conformational changes in TM6 leave a space next to TM3, which TM7 can occupy by spontaneously moving inwards, creating intracellular and extracellular conformational changes that affect other receptor regions such as the G protein binding-site or ECL2.”

On p36-37: “As such, there is a correlation between sustained binding of PG lipids to intracellular regions of TM6 and enhanced TM6 conformational change, resulting in more efficient G protein docking, rearrangement of the internal water-mediated polar network, tryptophan sidechain toggling, more stable protein-agonist H-bonding, and increased stabilization of ECL2. This creates a multidirectional allosteric network where different entities affect the action of others. ”

On p37: “In DOPG, ECL2 tends to be stabilized in a higher position above the orthosteric pocket than in PC membranes (S26 Fig), which appears to assist TM3 conformational change and vice versa, as well as enhancing the stability of bound agonist. This indirect relationship between membrane phospholipids and ECL2 may provide a structural explanation for how GPCRs exhibit different ligand efficacies in different cell lines.”

*23. Regarding the lipid interactions - I feel that a better characterization is required. Is the lipid always there? Are there other lipid binding sites?*

The protein-lipid interactions we show on the external surface of A2aR in the four identified intracellular DOPG hot spots (previously in S11 Fig) are remarkably specific relative to other non-specific interactions. Beyond these four hot spots, we do not observe any specific protein-lipid interactions of significance in a DOPG membrane. Based on comments by reviewer 2, this protein-lipid analysis is now grouped together in a new version of Fig 6 so that hot spots and specific protein-lipid interactions can be visualized together. Furthermore, we have narrowed these down to only display protein-lipid interactions that are the most stable, whilst previously we showed small groupings of lipids, which contained different degrees of physical stability. The DOPG lipids we now focus on are resident in these particular surface binding-sites for the longest time: commonly up to 2 microseconds or several hundreds of nanoseconds at least, as well as being observed across multiple replicas. We have generated a new analysis and supplementary figure for one of these specific intracellular protein-lipid interactions: close DOPG interaction with residue H6.32 on the external surface of TM6 (see new S13 Fig). Unlike the previously described interaction between a single DOPG molecule and ionic lock residue Arg3.50 in the intracellular G protein binding cavity of the receptor (which is not universal across all simulations), this surface TM6-DOPG interaction is present across all replicas but not always exclusively formed by any one single DOPG lipid throughout. As the membrane is dynamic, it is possible for one lipid to replace another whilst still maintaining the same overall binding mode and this type of lipid exchange can be observed in our simulations from time to time. We have therefore added the following comment to p21:

“Each of these protein-lipid interactions is observed across all replicas in a DOPG membrane and marked by particularly stable lipid binding modes. For example, in several trajectories, a single DOPG molecule closely interacts with residue H230 continuously for 2 µs, or in others, one lipid is exchanged for another after 500-1000 ns and the interaction is re-established (S13 Fig).”

*24. Line 491: “In addition, adenosine-bound A2aR in DOPG displays the same four protein-lipid allosteric interaction hot-spots as seen with the apo state in DOPG: at TM1- H8, TM3-4, TM5 and TM6 (Fig 6, S11 Fig).” >> In Figure S11, do the snapshots belong to apo-state or ligand bound state? Could the authors update the figure legend?*

In S11 Fig (now newly updated Fig 6), these structures refer to the adenosine-bound state. As these interactions are very similar in the apo state, we decided not to show them twice. We hope the reviewer agrees. We have also updated the figure legend accordingly in Fig 6.

*25. Line 776: “Interestingly, when comparing A2aR activation with that of CB1 [53], it is striking that activation of the latter proceeds more consistently and in different membrane types. This may be a feature of the type of agonist used in each study, for example a smaller and potentially weaker endogenous agonist here in A2aR, and a larger potentially stronger synthetic agonist in CB1”. >> If this statement is considered, then the lipid induced effect observed here may not be a universal phenomenon for this receptor. In that case, the title of the paper should explicitly mention the use of adenosine (natural agonist) as an agonist for the study.*

In order to properly test this hypothesis, we have performed extra MD simulations of A2aR with bound NECA, which is a bigger, synthetic and more potent agonist than adenosine, in quadruplicate and in DOPG and DOPC homogeneous membranes, respectively. We have included this extra data in Fig 10, as well as several extra supplementary figures (S19, S20, S21, S22, S23, S25, S26 Figs) and described our findings in a new section in Results, entitled: “NECA is a more potent agonist than adenosine.” (please, see p31-33). These new results with NECA show a very similar pattern to that with bound adenosine, which demonstrates consistent allosteric modulation of the receptor by DOPG lipids, mediated by stable protein-lipid interactions at specific intracellular locations, leading to full receptor activation in DOPG membranes but not in DOPC. Furthermore, due to the increased potency of NECA relative to adenosine, 3 of 4 replicas in DOPG with bound NECA obtain a fully active-like receptor state, while in DOPC, only an intermediate receptor conformation is obtained. In agreement with bound adenosine in a DOPG membrane, two distinct A2aR active-like receptor conformations are obtained with bound NECA: one of which is similar to the crystallized G protein-bound receptor conformation (PDB id: 6GDG), while the second appears to be distinct (see new Fig 10). These results show that receptor allosteric modulation by DOPG lipids is likely a universal mechanism that works for different A2aR agonists, natural or synthetic, weak or strong. Finally, in terms of comparison between adenosine A2A and CB1 receptors, it appears that their difference in activation in different membranes is due to the receptors themselves, not agonist size or potency. This is discussed on p42:

“Interestingly, when comparing A2aR activation with that of CB1, it is striking that activation of the former only proceeds with PG lipids while activation of the latter proceeds in both PG and PC lipid environments, albeit faster and more reliably with PG. This may be because these two receptors show different levels of activity. In particular, CB1 is known to have high constitutive activity and may be easier to activate relative to other GPCRs.”

And also on p42: “This could be one reason why activation of A2aR is observed more scarcely in the microsecond timescale relative to CB1 and why protein-lipid interactions appear to be so important for A2aR.”

*Reviewer #2: In this manuscript, Bruzzese at all., performed MD simulations of the A2A receptor inactive conformation in adenosine-bound and apo forms in DOPG and DOPC and observed transition to intermediate active receptor conformations in DOPC bound to adenosine and DOPG in apo and adenosine-bound forms. In the case of the adenosine-bound receptor form in DOPG they got a conformation that binds the Gs protein in docking studies. They showed that DOPG has positive allosteric effects into active conformation transition. The study is built on the available crystal structures of A2A receptors in inactive, active and intermediate conformations and uses a number of characteristic structural features of the receptor active state to monitor conformational changes. The manuscript is clearly written and the claims are convincingly supported. The work is recommended for publication after addressing the following comments:*

*1. The quality of the images in the main text is really poor, please check.*

The images uploaded to PLOS were high-resolution compressed TIFF files, whose quality is extremely high. We do not understand why these images appear so poor in the review PDF document generated by PLOS. We assume these issues arise because high-resolution images are not properly transferred by PLOS. We hope this will be fixed in later steps although we have no direct control over this.

*2. Page 17, line 353 ‘are these’ should be corrected. This manuscript would benefit from review by a professional editing service.*

We thank the reviewer for the advice. We have edited the manuscript as carefully as we can prior to re-submission. We have also changed the sentence in question on p18 to:

“However, these conformational changes are only maintained consistently over 2.0 μs in replica #3, while in replicas #1 and #4 such intermediate-like features are more transient and coincide only briefly (specifically at 1.9 μs and 1.2 μs, respectively).”

*3. Figure 6. Good to label helices to link with the discussed text on page 19-20.*

Agreed. Please see updated Fig 6.

*4. Page 28-29, units should be provided for interface docking score.*

The interface docking score generated by Rosetta does not have units. It is an arbitrary scale of 0 to minus 10 reflecting interaction quality. We now explain this more extensively on p33-34:

“Mainly, adequate docking is achieved due to proper separation of TM5/TM6 from TM3. The re-docking of the active crystal structure of A2aR with Gsα generates an interface docking score of -7.9 (no units, I_sc range from 0.0 to -10.0; more negative is better with -5.0 representing a threshold for respectable interaction).”

*5. Figure S1, S2. Dashed lines are shown to indicate the distances but the values are not shown. It is hard to appreciate the differences in distances.*

We have added distances next to dashed lines in both figures.

*6. Figure 11S should be in the text.*

We have combined S11 Fig with Fig 6 to make a new Fig 6, which is in the text.
